# *Fasciola hepatica* Cathepsin L Zymogens: Immuno-Proteomic Evidence for Highly Immunogenic Zymogen-Specific Conformational Epitopes to Support Diagnostics Development

**DOI:** 10.1021/acs.jproteome.2c00299

**Published:** 2022-07-18

**Authors:** Clare F. Collett, Helen C. Phillips, Maggie Fisher, Sian Smith, Caroline Fenn, Phil Goodwin, Russell M. Morphew, Peter M. Brophy

**Affiliations:** † Institute of Biological, Environmental and Rural Sciences, Aberystwyth University, Aberystwyth SY23 3DA, U.K.; ‡ Ridgeway Research Ltd., Park Farm Buildings, Park Lane, St. Briavels, Gloucestershire GL15 6QX, U.K.; § Bio-Check UK, Spectrum House, Llys Edmund Prys, St. Asaph Business Park, St. Asaph, Denbighshire LL17 0LJ, U.K.

**Keywords:** fasciolosis, diagnostics, cathepsin, recombinant, triclabendazole

## Abstract

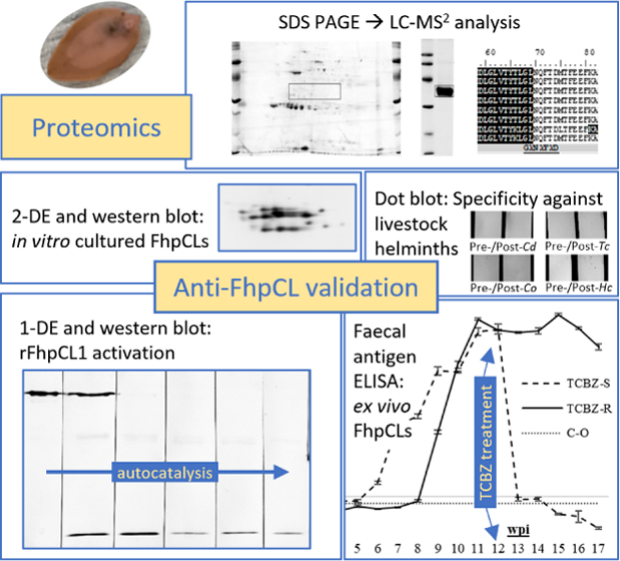

*Fasciola hepatica*, the common liver fluke and causative agent of zoonotic fasciolosis, impacts on food security with global economic losses of over $3.2 BN per annum through deterioration of animal health, productivity losses, and livestock death and is also re-emerging as a foodborne human disease. Cathepsin proteases present a major vaccine and diagnostic target of the *F. hepatica* excretory/secretory (ES) proteome, but utilization in diagnostics of the highly antigenic zymogen stage of these proteins is surprisingly yet to be fully exploited. Following an immuno-proteomic investigation of recombinant and native procathepsins ((r)FhpCL1), including mass spectrometric analyses (DOI: 10.6019/PXD030293), and using counterpart polyclonal antibodies to a recombinant mutant procathepsin L (anti-rFhΔpCL1), we have confirmed recombinant and native cathepsin L zymogens contain conserved, highly antigenic epitopes that are conformationally dependent. Furthermore, using diagnostic platforms, including pilot serum and fecal antigen capture enzyme-linked immunosorbent assay (ELISA) tests, the diagnostic capacities of cathepsin L zymogens were assessed and validated, offering promising efficacy as markers of infection and for monitoring treatment efficacy.

## Introduction

Many parasitic helminths of medical and veterinary importance utilize cysteine proteases as virulence factors for invasion, nutrition, and immune evasion.^1−5^ However, the common liver fluke, *Fasciola hepatica*, has the largest family of these proteins, consisting of 17 cathepsin L (CL) cysteine proteases within clades 1–5 and three developmentally regulated, juvenile-specific cathepsin B isotypes.^6−8^ The diverse functionality and pathogenicity of *F. hepatica* CLs are well-documented^2,9,10^ and consequently, CL proteases and associated derivatives have been key targets for fasciolosis vaccines^11−13^ and diagnostics.^14−16^

The activation process of CL proteases involves three protein stages and begins within fluke gastrodermal lysosomes from which nascent zymogens (pre-procathepsins) are guided *via* the signal peptide (pre-peptide) to the epidermis and gut lumen within secretory vesicles.^17^ Following lumen entry of procathepsins, autocatalytic processing commences within this low pH environment, leading to inhibitor (pro-) peptide cleavage,^18,19^ and during frequent fluke regurgitations of digesta, activated CL proteases are released into the host extracellular matrix.^17,20^ Though highly biochemically stable, it has been determined that the small, acidic pH range reflective of the fluke gut lumen is optimal for clade 1, 2, and 5 CL proteases to digest host hemoglobin and albumin for gastrodermal peptide absorption.^19,21,22^ However, CL proteolytic activities continue despite vomitus expulsion into the extracellular matrix at physiological pH, whereupon the proteases readily digest host interstitial tissue and immunoglobulins.^23−26^ Consequently to their prolific excretion, cathepsin proteases comprise the main parasite proteomic component recovered from *F. hepatica* infection and culture, including adult CLs from bile extracts *ex vivo*(27) and both adult CLs and juvenile cathepsins within excretory–secretory (ES) products derived *in vitro.*(28,29)

In keeping with their overabundance and immunogenicity in *F. hepatica* ES products,^30^ CL proteases represent a key diagnostic target for fasciolosis. MM3 monoclonal antibodies raised to the adult fluke 13–25 kDa ES subproteome fraction, containing CL proteases,^27,31^ form the basis of Bio-X enzyme-linked immunosorbent assay (ELISA) kits (BIO K201 and K211 tests; Bio-X Diagnostics, Jemelle, Belgium), which are also validated for the diagnosis of anthelmintic sensitivity and treatment success.^32,33^

Though MM3 recognition of endogenous and recombinant CL epitopes has been confirmed,^15,30^ there is no evidence for MM3-procathepsin L binding activity, thought to be caused by antigen conformational differences.^30^ Despite this, the antigenic propensity of the complete CL protein sequence has been mapped, identifying both protease- and zymogen-specific epitopes with immunogenic potential, and as such, peptide derivatives predominantly from the protease region have been tested toward alternative options for fasciolosis diagnosis^34−36^ and protection.^37^ Despite predictions of antigenicity of zymogen oligomers, the abundance and established immunoreactivity of CL protease epitopes with host serum and MM3 has precluded focus on zymogen-specific epitopes for diagnostic consideration. However, signal peptides and certain inherent residues have demonstrably high immunogenicity, both prior to and after cleavage from the parent protein, which has hindered their prospective and growing applications in diagnostics, vaccines, and molecular biology techniques.^38−42^ Alongside the pre-peptide, the CL pro-peptide has also demonstrated immunodominance in procathepsin (pCL)-mediated protection.^43^ Thus, the aim of this work was to determine the diagnostic utility of CL zymogens, specifically inhibitor peptide epitopes.

## Experimental Procedures

### Recombinant (Pre)Procathepsin L (p/pCL) Zymogens

Two purified recombinant procathepsin L1 proteins (expressed in *Pichia pastoris* GSII5 yeast) were kindly gifted by Professor Dalton (Galway, Ireland), including a wild-type (rFhpCL1_WT_) with the capacity for protease activation and a mutant designed to prevent autocatalytic pro-peptide cleavage (rFhΔpCL1; Leu12Pro at pro-peptide C-terminus; amino acid (aa) 95 *in situ*).^18,44^ rFhpCL1_WT_ activation and pro-peptide cleavage were conducted based on the protocol by Stack et al.,^44^ initiated using activation buffer (0.1 M sodium citrate, pH 5.0; 2 mM dithiothreitol; 2.5 mM ethylenediaminetetraacetic acid), incubated at 37 °C for 0, 30, 60, 90, or 120 min and stopped on ice. A purified, refolded *F. hepatica* procathepsin L (rFhpCL1, expressed in *Escherichia coli* M15 (pREP4) bacteria) was also kindly provided by Doctor Martínez-Sernández (Universidad de Santiago de Compostela, Spain).^30^

### Isolation of *F. hepatica* ES Products

Live *F. hepatica* were collected at a local abattoir from freshly slaughtered sheep livers with naturally acquired infections. Adult *F. hepatica* were prepared for *in vitro* maintenance, and whole ES products, reflective of live and terminated flukes, were obtained, as described by Morphew et al.^27^ Briefly, size-matched adults (1–3 cm length) were selected, and replicates of 10 flukes were grouped for *in vitro* maintenance directly (live) or after termination (dead) in ethyl 4-aminobenzoate (Sigma-Aldrich, U.K.; 1% (w/v) in ethanol (Fisher Scientific, U.K.)), with 3 mL of fresh supplemented culture medium per fluke and incubation at 37 °C. For the extraction of whole ES products, media supernatants were clarified by 300*g* centrifugation and precipitated *via* the TCA method, as previously described.^27^

### Animal Samples

#### Infection Sera and Fecal Sample Preparation

All sera and fecal samples were generated by Ridgeway Research Limited (St Briavels, U.K.), isolated from sheep and cattle experimentally infected with fluke (*F. hepatica*; *Calicophoron daubneyi*) or nematode (*Haemonchus contortus*; *Teladorsagia circumcincta*; *Cooperia oncophora*) helminths. Sera and fecal samples were obtained at weekly intervals between at most 0–17 weeks post infection (wpi) and subsequently stored at −20 °C (fecal samples) or −80 °C. Crude feces were homogenized by inversion and vortexing in distilled water (UV-sterilized; 15 MΩ) at a ratio of 1:3 (water/feces) and then centrifuged at 1000–5000*g* at 4 °C for at least 10 min until pelleted and stored at −20 °C. Further samples were obtained from experimental infections with one of three strains per sheep of either TCBZ-susceptible (TCBZ-S) or -resistant (TCBZ-R) *F. hepatica*, involving clinically administered TCBZ treatment (10 mg/kg) at 12 wpi. Representative samples for each time point and phenotype were achieved by pooling fecal supernatants (TCBZ-S strains: Aberystwyth, Italian; TCBZ-R strains: Kilmarnock and Stornoway) and whole sera (TCBZ-S: Aberystwyth, Italian, Miskin, excluding 17 wpi Aberystwyth sera; TCBZ-R: Kilmarnock, Penrith, Stornoway).

#### Anti-rFhpCL1 Polyclonal Sera and IgG Purification

Purified recombinant *F. hepatica* procathepsin L1 (rFhΔpCL1) antigen (Ag) was used to raise polyclonal serum antibodies (PcAb) in two laboratory rabbits (Lampire Biological Laboratories). Immunizations with approximately 0.3 mg Ag mixed with an equal volume of complete or incomplete Freunds’ adjuvant (CFA/IFA) were given at day one (Ag-CFA), 21 (Ag-IFA), and 42 (Ag-IFA), with the first two *via* the popliteal lymph node following Evan’s blue introduction and the final booster by intradermal injection. Blood samples were collected at pre-immunization (day 0) and post-immunization after 50 days, from which whole sera were isolated and pooled per collection day, and sera were stored at −80 °C until required.

Purification of IgG from pre- and post-immunization PcAb samples was conducted using protein A affinity chromatography as per the manufacturer’s guidelines (ABT, Web Scientific, U.K.). Briefly, protein A-coated beads were equilibrated in binding buffer (25 mM sodium phosphate, pH 7.0) before applying sera diluted 1:1 in binding buffer for 45–60 min. The flow-through was collected, and the resin was washed with binding buffer until the flow-through *A*_280_ was equal to the binding buffer *A*_280_ and then IgGs were eluted (glycine 100 mM, pH 3.0) and neutralized (1 M tris, pH 9.0) as per the manufacturer’s recommendation. Protein A-purified IgG sample elutants were concentrated using Amicon Ultra 3K centrifugal filters (Merck, U.K.) according to the manufacturer’s protocol, conducted at 4 °C and 14,000*g* for 30 min. Samples were washed in storage buffer (0.05% sodium azide (w/v) in PBS (0.1 M, pH 7.4; Sigma-Aldrich, U.K.)), centrifuged as before, and resuspended in storage buffer. For IgG biotinylation, purified post-immunization IgGs were labeled using the Lightning Link rapid biotinylation kit (Innova Biosciences, U.K.) according to the manufacturer’s instructions. Briefly, purified IgGs were incubated with biotin at 20 °C for a minimum of 2 h and a maximum of 14 h before reactions were stopped, and biotinylated antibodies were stored at 4 °C until required.

### Proteomics and Western Hybridization

For 1-D (1-DE) and 2-D (2-DE) sodium dodecyl sulfate polyacrylamide gel electrophoresis (SDS PAGE), protein samples were prepared and electrophoresed as previously described,^45^ as specified per lane/gel in this study. Gels destined for direct examination or mass spectrometry were fixed (10% (v/v) acetic acid; 40% (v/v) ethanol), washed (H_2_O, 18 MΩ), and then stained with Coomassie blue (PhastGel Blue R, Amersham Biosciences, U.K.) as per the manufacturer’s instructions and destained in acetic acid (1% (v/v)) as required. For liquid chromatography-tandem mass spectrometry (LC-MS^2^), technical replicate (duplicate) gel pieces were excised, prepared, and analyzed as previously described,^45,46^ except for the use of a HPLC Prot-ID Chip (Agilent 6550 iFunnel Q-TOF, Agilent Technologies, U.K.). Where necessary for complex protein mixtures, samples were analyzed using an Orbitrap Fusion Tribrid mass spectrometer (Thermo Scientific, U.K.) coupled to an UltiMate 3000 liquid chromatography tower (Dionex, Thermo Scientific, U.K.) and Zorbax Eclipse Plus reversed-phase C18 column at 30 °C (Agilent Technologies, U.K.) operated as follows. Mobile phases for gradient elution were maintained at a flow rate of 0.1 mL/min using ultrapure water (18.2 MΩ) with 0.1% formic acid (Fluka, U.K.) (eluent 1) and 95:5 acetonitrile (Optima, Fisher Scientific, U.K.): ultrapure water with 0.1% formic acid (eluent 2). The initial condition was 3% eluent 2 with a linear increase to 40% over 9 min, increasing to 100% eluent 2 in a further 2 min, and then held for 1 min at 100% eluent 1 before equilibration at initial conditions for a further 1.5 min. Ions were generated using a heated ESI source at 3500 V in positive mode, sheath gas at 25 °C, aux gas at 5 °C, a vaporizer temperature of 75 °C, and an ion transfer temperature of 275 °C. Standard peptide analysis parameters were used comprising a data-dependent MS^2^ experiment, whereby parent ions were detected in profile mode in the 375–1500 *m*/*z* range in the Orbitrap at a resolution of 120,000 and maximum injection duration of 50 ms in positive mode. MS^2^ data were then collected in data-dependent mode, including charge states of 2–7 and dynamic exclusion of masses for 20 s after initial selection for MS^2^. Ions were formed by fragmentation by collision-induced dissociation with a collision energy of 35%, and resulting ions were detected in the ion trap in centroid mode. Data files were assessed using the MASCOT MS^2^ ions search (Matrix Science) against the GenBank database (v204), with the search parameters set as previously described,^45,46^ except for the inclusion of error tolerance and exclusion of a decoy search tool. The mass spectrometry proteomics data were deposited to the ProteomeXchange Consortium *via* the PRIDE partner repository with the dataset identifier PXD030293 (DOI: 10.6019/PXD030293),^47^ and details of sample nomenclature are available in the Supporting Information (Supporting Table S1).

For western hybridization procedures, 1- and 2-DE-separated samples were transferred to nitrocellulose membrane (NCM 0.45 μm; GE Healthcare, U.K.), which was confirmed by Amido Black staining, and membranes were prepared as previously described,^48^ with antibodies tested as follows. Whole anti-rFhΔpCL1 sera were diluted as required for each application and incubated with membranes at room temperature for an hour prior to incubation with 1:30,000 diluted anti-rabbit IgG-AP secondary antibodies (A3687, Sigma-Aldrich, U.K.) and detected using the BCIP-NBT system and imaged using a Bio-Rad GS-800 calibrated densitometer (Bio-Rad, U.K.) as previously described.^45^ Uncropped images of entire membranes from all western hybridization procedures are provided in the Supporting Information (Supporting Figure S1).

#### Procathepsin L-Based Immunogenicity Predictions

LC-MS^2^-confirmed FhpCL protein sequences from recombinant procathepsin L 1-DE samples and 2-DE-separated *F. hepatica* ES were aligned using Clustal O (clustalo). Antibody and B cell epitopes were predicted using the Kolaskar and Tongaonkar method^49^ with tools by the Immune Epitope Database and Analysis Resource (iedb.org) and the Immunomedicine Group (imed.med.ucm.es, Universidad Complutense de Madrid).

### Enzyme-Linked Immunosorbent Assays (ELISAs) and Statistics

#### Direct ELISA for the Detection of Anti-rFhΔpCL1 Serum IgG

rFhΔpCL1 in 100 μL/well coating buffer ([0.5 μg/mL] 0.1 M NaHCO_3_–Na_2_HCO_3_ pH 9.5) was coated onto Immulon 4HBX plates (Thermo Scientific, U.K.) overnight at 4 °C, then blocked with 200 μL/well blocking buffer (2% bovine serum albumin (BSA, SRE00036, Sigma-Aldrich, U.K.)) in PBS-Tween-20 (PBS-T; PBS: pH 7.4; P4417, Sigma-Aldrich, U.K.; with 0.05% Tween-20 (Fisher Scientific, U.K.)). Subsequently, 100 μL/well 1:750 pooled sera samples in 1% BSA-PBS-T were incubated, followed by 100 μL/well 1:30,000 anti-sheep IgG secondary antibody (A5187, Sigma-Aldrich, U.K.) in 1% BSA-PBS-T and then detection with 100 μL/well pNPP substrate solution (P7998, Sigma-Aldrich, U.K.). AP-pNPP reactions were stopped after 30 min by the addition of 25 μL/well 3 M (N) NaOH and OD values were read at 405 nm. All steps were incubated for 1 h at 37 °C, and washing steps were included before and after all steps, using 200 μL/well PBS-T five times (1 min each) with agitation. Average OD values were calculated by subtracting OD values of wells coated with irrelevant Ag (0.05% BSA) from OD values of wells coated with rFhΔpCL1, with overall OD measurements averaged between two duplicate measurements conducted on two different days.

#### Sandwich ELISA for Fecal FhpCL1 Antigen Capture

Polyclonal anti-rFhΔpCL1 IgG and polyclonal IgG from a nonimmunized rabbit in 100 μL/well coating buffer [5 μg/mL] were coated onto Immulon 4HBX plates overnight at 4 °C, then blocked with 200 μL/well 2% BSA-PBS-T blocking buffer. Subsequently, 100 μL/well of pooled fecal samples per experimental parasite or *F. hepatica* TCBZ-S/-R strain infection were incubated, then detected with 100 μL/well 1:25,000 anti-rFhΔpCL1 IgG-Biotin in 1% BSA-PBS-T, followed by 100 μL/well avidin–peroxidase (A3151, Sigma-Aldrich, U.K.) in PBS-T. All Ag and antibody steps were incubated for 1 h at 37 °C, and avidin–peroxidase was incubated for 30 min at 37 °C. Washing steps were included before and after all steps as previously described. For final detection, 100 μL/well 1-Step Ultra TMB-ELISA solution (34028, Thermo Scientific, U.K.) was incubated in the dark at room temperature (≈ 20 °C) for 5 min and stopped using 100 μL/well 2 M H_2_SO_4_. OD of wells was measured at 450 nm, and average measurements were calculated by subtracting OD values of wells coated with nonimmunized rabbit IgG from OD values of wells coated with anti-rFhΔpCL1 rabbit IgG, with overall OD measurements averaged between two duplicate measurements conducted on two different days.

### Statistical Analyses

Cutoff values were calculated as 1 standard deviation above the mean sample OD value of the negative control (irrelevant antigen/uninfected sample), which were calculated per assay, as previously described.^15^

### Dot Blots

For dot blots, NCM was washed with distilled water, equilibrated in Bjerrum buffer (25 mM (w/v) tris, pH 8.3; 192 mM (w/v) glycine; 20% (v/v) methanol), and then dried and allowed to acclimatize to room temperature. Then, 0.01 μg rFhΔpCL1 antigen resuspended in 2 μL PBS was applied to absorb onto the membrane, and then the blots were allowed to dry at room temperature and thereon treated as in the western blotting procedure. Each antigen sample dot was incubated with uninfected or infected sera, where *C. daubneyi*, *T. circumcincta*, or *H. contortus* sheep infection sera were diluted to 1:700 and detected with 1:30,000 anti-sheep IgG-AP secondary antibody, and *C. oncophora* cattle infection sera were diluted to 1:100 and detected with 1:30,000 anti-bovine IgG-AP secondary antibody (A0705, Sigma-Aldrich, U.K.). A positive reaction was included by diluting anti-rFhΔpCL1 sera to 1:5000 and detected with 1:30,000 anti-rabbit IgG-AP secondary antibody.

## Results and Discussion

### Comparative Antigenicity of Recombinant *F. hepatica* Procathepsin Ls

Cathepsin L (CL) proteases are in dominant abundance in juvenile and adult fluke ES products^2,27,31^ as a consequence of their multifaceted roles in fluke nutrition, pathogenesis, and immune evasion.^19,50−52^ Despite the long-standing consideration of CL proteases as diagnostic and vaccine candidates for fasciolosis control,^11,53,54^ there is evidence to support the highly antigenic propensity of CL zymogens. We sought to explore this through the evaluation of three recombinant CL zymogens and representative *in vitro* native equivalents, confirming protein identity and subsequently assessing their antigenicity.

**Figure 1:**
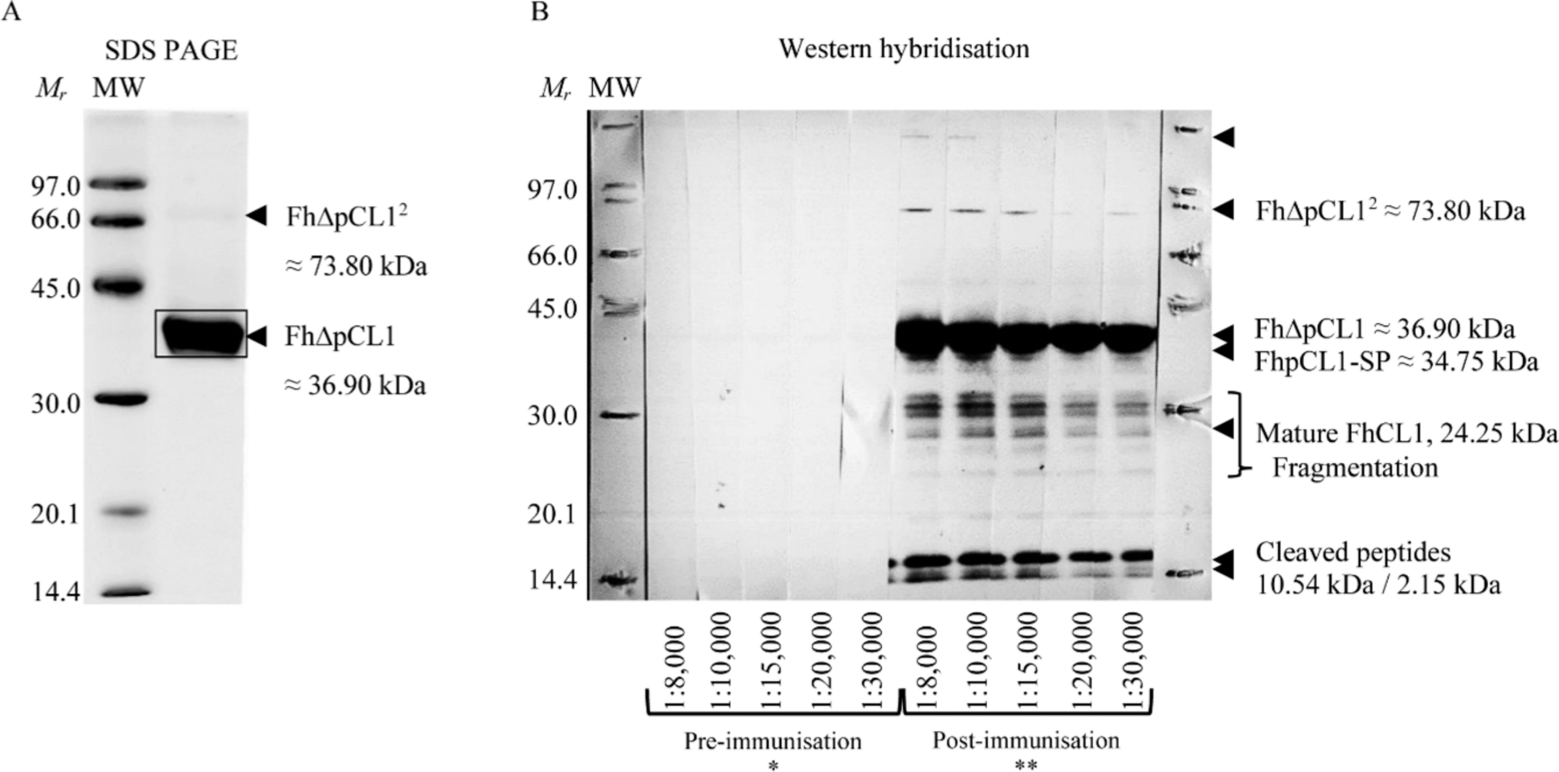
1-DE of recombinant mutant *F. hepatica* procathepsin L1 (rFhΔpCL1) and immunoreactivity against polyclonal anti-rFhΔpCL1 IgG. (A) 2 μg rFhΔpCL1 was analyzed by 1-DE, and the intact zymogen fragment (boxed) was excised and analyzed by LC-MS^2^ (Table 1). Two hits were consistent between duplicate sample submissions (procathepsin L1 chain A, 2O6X_A; cathepsin L-like proteinase, ADP09371.1), including peptide recovery from pro-peptide (16–105 aa) and cathepsin L protease (106–326 aa/TERM) regions. (B) 1 μg rFhΔpCL1 was probed with 1:800–1:30,000 pre- (*) and post-immunization (**) rabbit sera and detected by alkaline phosphatase-conjugated anti-rabbit IgG raised in goat. Abbreviations: MW, Amersham Low Molecular Weight SDS Calibration Kit (Mr); FhΔpCL1^2^, dimer-sized protein; and FhpCL1-SP, procathepsin with cleaved signal peptide.

An intact recombinant mutant procathepsin L1 (rFhΔpCL1; Leu-Pro C-terminal pro-segment substitution; L95P *in situ*)^44^ was separated by 1-DE, and LC-MS^2^ analysis of the zymogen-containing gel section (36.9 kDa; Figure 1A: boxed) identified two *F. hepatica* protein hits (Table 1), including procathepsin L1 chain A (GenBank: 2O6X_A) and cathepsin L-like proteinase (GenBank: ADP09371.1). Further hits were identified based on peptide samesets, subsets, and intersections, which are summarized in the Supporting Information, including the top hits in bold (Supporting Table S2: rFhΔpCL1). Average sequence coverage of the top two hits identified the recovery of peptides pertaining to both pro-segment pro-peptide (16–105 aa) and protease (106–326 aa) regions (average sequence coverage: 2O6X_A, 73.0 ± 10.0%; ADP09371.1, 39.0 ± 8.0%), confirming the presence of inhibitor peptide, protease, and overlapping, intact inhibitor–protease regions of the antigen. A sequence alignment (Supporting Figure S2A) identified 10 residue differences within the protease region (2O6X_A *versus*ADP09371.1: Gly116Cys; Gln166Glu; Thr182Arg; Phe202Tyr; Arg237Ser; Ser238Gly; Arg250Gly; Val251Leu; Val288Ala; Pro304Leu) in addition to the absence of the signal peptide from 2O6X_A (1–15 aa). A BLAST search was used to identify a protein familial clade for rFhΔpCL1, and the highest-scoring common hit was identified as the secreted cathepsin L1 (GenBank: AAB41670.2), with 99.0 and 97.0% identity, respectively. As per the CL protease clade organization detailed by Morphew et al.^28^ and AAB41670.2 classification as a CL1A, rFhΔpCL1 was putatively assigned to the cathepsin L1A clade.

**Table 1:** LC-MS^2^ Identification of 1-DE-Separated Recombinant *F. hepatica* CL Zymogens^a^

		MS/MS ion search					highest-scoring GenBank hit			
				peptides matched (non-duplicate)	sequence coverage					
recombinant procathepsin L sample	approximate molecular weight (sample number)	GenBank hit	MASCOT score (Av)	peptides matched (non-duplicate)	average percentage coverage (%)	collective residue coverage (aa)	protein	organism	accession	*E*-value
rFhApCLl	37	gi|163310848	1677.0 ± 1078.0	71.5 ± 37.5	73.0 ± 10.0	15–24, 42–282, 292–310	Chain A, Crystal Structure Of Procathepsin L1 (1–310)	*F. hepatica*	2O6X A	0.0
		gi|310751866	441.5 ± 281.5	30.5 ± 17.5	39.0 ± 8.0	31–40, 58–124, 151–185, 206–230, 289–298, 308–324	Cathepsin L-like proteinase (1–326)	*F. hepatica*	ADP09371.1	0.0
rFhpCL1WT	37	gi|116488416	132.5 ± 56.5	17.0 ± 4.0	38.5 ± 1.5	91–124, 186–205, 215–230, 254–298, 308–324	Secreted cathepsin L1 (1–326)	*F. hepatica*	AAB41670.2	0.0
	35	gi|116488416	117.0 ± 51.0	13.5 ± 3.5	41.0 ± 4.0	66–81, 91–124, 186–205, 215–230, 254–298, 308–324	Secreted cathepsin L1 (1–326)	*F. hepatica*	AAB41670.2	0.0
rFhpCLl	37 (1)	gi379991182	90.5 ± 9.5	10.5 ± 3.5	30.0 ± 4.0	58–83, 91–106, 186–205, 215–230, 289–298, 308–324	Cathepsin protein CatL1-MM3p, partial (1–326)	*F. hepatica*	CCA61803.1	0.0
	32 (2)	gi|379991182	101.0 ± 29.0	14.0 ± 4.0	36.5 ± 7.5	58–81, 91–147, 186–205, 215–230, 289–298, 308–324	Cathepsin protein CatL1-MM3p, partial (1–326)	*F. hepatica*	CCA61803.1	0.0
	28 (3)	gi|379991182	479.0 ± 23.0	40.5 ± 0.5	62.5 ± 0.5	58–81, 91–147, 186–205, 215–230, 263–298, 308–324	Cathepsin protein CatL1-MM3p, partial (1–326)	*F. hepatica*	CCA61803.1	0.0
		gi|310751866	245.0 ± 20.0	24.0 ± 1.0	42.5 ± 0.5	58–81, 91–150, 215–230, 263–288, 306–324	Cathepsin L-like proteinase (1–326)	*F. hepatica*	ADP09371.1	0.0
	24 (4)	gi|379991182	148.0 ± 24.0	18.5 ± 4.5	45.0 ± 1.0	58–81, 91–205, 215–230, 289–298, 308–324	Cathepsin protein CatL1-MM3p, partial (1–326)	*F. hepatica*	CCA61803.1	0.0
	18 (5)	gi|379991182	270.5 ± 32.5	20.5 ± 1.5	44.0 ± 0.0	58–83, 91–147, 186–205, 215–230, 289–298, 308–324	Cathepsin protein CatL1-MM3p, partial (1–326)	*F. hepatica*	CCA61803.1	0.0
	≤14 (6)	gi|379991182	462.0 ± 16.0	40.5 ± 3.5	51.0 ± 0.0	58–83, 91–147, 186–205, 215–230, 270–298, 308–324	Cathepsin protein CatL1-MM3p, partial (1–326)	*F. hepatica*	CCA61803.1	0.0
		gi|310751866	203.0 ± 35.0	27.0 ± 0.0	42.0 ± 0.0	58–147, 215–230, 269–288, 306–324	Cathepsin L-like proteinase (1–326)	*F. hepatica*	ADP09371.1	0.0
		gi|19909509	123.5 ± 18.5	16.0 ± 2.0	28.0 ± 0.0	107–115, 125–147, 186–205, 265–286, 306–322	Cathepsin L (1–324)	*F. gigantica*	BAB86959.1	0.0

a Recombinant mutant (rFhΔpCL1) and wild-type (rFhpCL1WT) procathepsin L (Ireland) and a second recombinant procathepsin L (Spain) were analyzed by duplicate 12.5% SDS PAGE, and bands of interest were selected for investigation using LC-MS^2^. Protein hits are shown following identification against the GenBank database (v204) using an in-house MASCOT (Matrix Science) server, with consistent hits reported with average scores between duplicate sample submissions. Significant hits identified with an average score of 67 or greater (*P* < 0.05) are shown, including reliable error tolerance and reporting significant hits consistent between duplicate sample submissions. Further hits based on peptide samesets, subsets, and intersections are available in the Supporting Information: Supporting Table S2.

Diagnostic applications of monoclonal antibodies, such as MM3,^14^ have advantages owing to the predetermined specificity for a selected epitope. In our approach, however, we sought to test the functionality and diagnostic utility of polyclonal antibodies so as to include multiple target epitopes of the *F. hepatica* procathepsin zymogen. As such, anti-rFhΔpCL1 polyclonal sera were raised and optimal working titers were determined using western hybridization of pre- and post-immunization sera (1:8000–1:30,000 diluted) against 1-DE-separated rFhΔpCL1 (Figure 1B). Western hybridization also confirmed the absence of reactive IgG in the pre-immunization sera and the presence of anti-rFhΔpCL1 IgG in post-immunization sera that were highly reactive to the intact 37 kDa antigen. Further proteins were also detected by this western hybridization that were not visible by 1-DE gel Coomassie staining (Figure 1) or Amido black NCM staining (Supporting Figure S3), including protein and peptide forms at approximately 75, 25–37, and ≤14 kDa consistent with dimers (FhΔpCL1^2^), intermediates, fragments, and inhibitor and signal peptides (10.58 and 2.15 kDa expected molecular weights, respectively).

**Figure 2:**
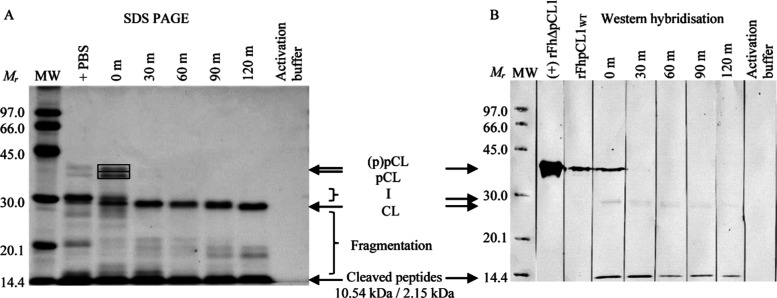
1-DE of recombinant wild-type *F. hepatica* procathepsin L1 (rFhpCL1^WT^) and immunoreactivity against polyclonal anti-rFhΔpCL1 IgG. (A) 5 μg of rFhpCL1^WT^ was analyzed by 1-DE, including inactivated protein and following autocatalysis into cathepsin L protease and cleaved pro-enzyme peptides. Two rFhpCL1^WT^ zymogen fragments of approximately 35 and 37 kDa (boxed) were excised and analyzed by LC-MS^2^ (Table 1), confirming one hit consistent between duplicate analysis (secreted cathepsin L1, AAB41670.2). (B) 0.5 μg rFhpCL1^WT^ from each autocatalysis time point was probed with 1:15,000 anti-rFhΔpCL1 polyclonal rabbit sera alongside 0.5 μg rFhΔpCL1(+) and detected by alkaline phosphatase-conjugated anti-rabbit IgG raised in goat. Abbreviations: MW, Amersham Low Molecular Weight SDS Calibration Kit (Mr); (p)pCL, (pre-)procathepsin L; I, intermediates proteins; and CL, cathepsin L protease.

rFhpCL1_WT_, a wild-type equivalent to rFhΔpCL1, was analyzed by 1-DE, and subsequently, western hybridization for direct comparison to the mutant antigen. Before, during, and after autocatalysis (Figure 2A), separation of the zymogen protein was demonstrated, leading to fractionation of peptides (<20 kDa), intermediates (24.25–34.75 kDa), and mature enzymes (24.25 kDa). LC-MS^2^ analysis of gel pieces containing protein either pre- (≈ 37.0 kDa, intact) or post- (≈ 35.0 kDa, intermediate) autocatalysis (Figure 2A: boxed) led to the identification of the secreted cathepsin L1 (GenBank: AAB41670.2) as the highest-scoring hit for both samples (Table 1). Further hits were identified based on peptide samesets, subsets, and intersections, which are summarized in the Supporting Information, including the top hits in bold (Supporting Table S2: rFhpCL1_WT_). Peptide recovery from both fractions also indicated sequence coverage of the top hits pertaining to pro-peptide, protease, and overlapping (inhibitor–protease) regions (average sequence coverage: intact ≈ 37 kDa zymogen, 38.5 ± 1.5%; intermediate ≈ 35 kDa protein, 41.0 ± 4.0%). Thus, as per the mutant pCL, rFhpCL1_WT_ was also putatively allocated to the cathepsin L1A familial clade.

The antigenic contribution of the rFhΔpCL1 and rFhpCL1_WT_ zymogen protein epitopes was assessed *via* the regulated autocatalysis of rFhpCL1_WT_ and immunoreactivity with anti-rFhΔpCL1 IgG *via* western hybridization. Antibodies bound almost exclusively to zymogen-specific epitopes at pro-enzyme and peptide-sized fractions in rFhpCL1_WT_ (Figure 2B), including at the intact zymogen and following autocatalysis and peptide fractionation (expected molecular weights of cleaved peptides: inhibitor, 10.58 kDa; signal, 2.15 kDa). Moreover, there was minor binding to intermediary and protease proteins, thus strongly suggesting that potent immunogenicity of the intact rFhpCL1 antigen is at inhibitor and/or signal peptide epitopes, possibly including pro-enzyme conformational epitopes.

**Figure 3:**
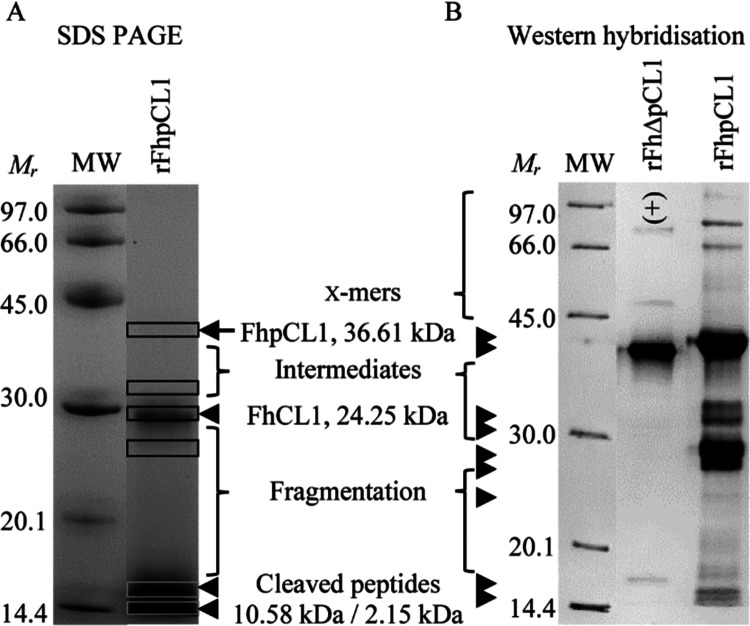
1-DE of recombinant *F. hepatica* procathepsin L1 (rFhpCL1) and immunoreactivity against polyclonal anti-rFhΔpCL1 IgG. (A) 20 μg rFhpCL1 was analyzed by 1-DE, and six protein fragments (boxed: 1–6) between ≈14 to 37 kDa were excised and analyzed by LC-MS^2^ (Table 1). One hit was consistent in all fragments for cathepsin protein CatL1-MM3p partial (CCA61803.1), and a further hit was found for fragments 3 and 6 (cathepsin L-like proteinase, ADP09371.1) and fragment 6 (cathepsin L, BAB86959.1) only. Peptide recovery between CCA61803.1 and ADP09371.1 hits included pro-peptide (16–105) and cathepsin L protease (106–326) regions, whereas BAB86959.1 peptides pertained to the protease (106–324) region only. (B) 2 μg rFhpCL1 was probed with 1:10,000 anti-rFhΔpCL1 polyclonal rabbit sera alongside 0.05 μg rFhΔpCL1(+) and detected by alkaline phosphatase-conjugated anti-rabbit IgG raised in goat. Abbreviations: MW, Amersham Low Molecular Weight SDS Calibration Kit (Mr); x-mers, dimer- and trimer-sized proteins.

To determine and compare the antigenicity of a different recombinant procathepsin L antigen from the mutant and wild-type rFhpCL1A (rFhΔpCL1/rFhpCL1_WT_), we tested a refolded native recombinant procathepsin L1 (rFhpCL1) purified under denaturing conditions, which was kindly provided by Doctor Martínez-Sernández (Universidad de Santiago de Compostela, Spain). Analysis by 1-DE indicated rFhpCL1 underwent autonomous autocatalytic processing and/or fragmentation prior to or upon dithiothreitol denaturation for SDS PAGE analysis, whereby six major fragments were determined (Figure 3A: boxed, approximate kDa: 37 (1), 32 (2), 28 (3), 24 (4), 18 (5), and ≤14 (6)) and analyzed by LC-MS^2^ for confirmation of protein identity. The highest-scoring and consistent *F. hepatica* protein result for all rFhpCL1 fragments was cathepsin L protein CatL1-MM3p partial (GenBank: CCA61803.1), followed by the cathepsin L-like proteinase (GenBank: ADP09371.1) in samples 3 and 6, and the cathepsin L (GenBank: BAB86959.1) in sample 6. Further hits were identified based on peptide samesets, subsets, and intersections, which are summarized in the Supporting Information, including the top hits in bold (Supporting Table S2: rFhpCL1). Data also indicated the presence of peptides matching the pro-peptide, protease, and overlapping (inhibitor–protease) regions of CCA61803.1 (30.0–62.5%, 44.83% average sequence coverage; all fragments) and ADP09371.1 (42.0–42.5%, 42.25% average sequence coverage; fragments 3 and 6), and the protease region only of BAB86959.1 (28.0% average sequence coverage, fragment 6) were detected. Since CCA61803.1 and ADP09371.1 isoforms are not yet assigned to a CL clade,^28^ the closest GenBank CL sequence assigned to a CL clade was identified (Supporting Figure S2B: AAR99519.1, 95 and 94% sequence identity, respectively), and consequently, rFhpCL1 was assigned to the CL1A clade.

rFhpCL1 antigenicity against anti-rFhΔpCL1 polyclonal sera was tested *via* western hybridization, whereby multiple rFhpCL1 protein fragments of ranging molecular weights retained reactive epitopes (Figure 3B), including at pro-enzyme (pCL), intermediates (I), and inhibitor and signal peptide-sized fractions (expected molecular weight of cleaved peptides: inhibitor, 10.58 kDa; signal, 2.15 kDa). Further evidence of immunoreactivity at approximately protease-sized (CL1) and further fragmented protein (F) bands was also detected, indicating further immunogenic peptides in rFhpCL1 and/or more epitope exposure following this degree of fragmentation.

### Recovery and Detection of Native Procathepsins from *In VitroF. hepatica* Culture

Increased antigen abundance following parasite activities and secretions are favorable in diagnostics where host immune exposure or direct antigen recovery can be detected. Consequently, many studies investigating flukicide- and death-induced changes in fluke ES proteome profiles have elucidated novel and immunogenic biomarkers.^27,56,57^ Thus, we sought to determine the presence and antigenicity of native *F. hepatica* CL zymogens from *in vitro* liver fluke cultures.

**Figure 4:**
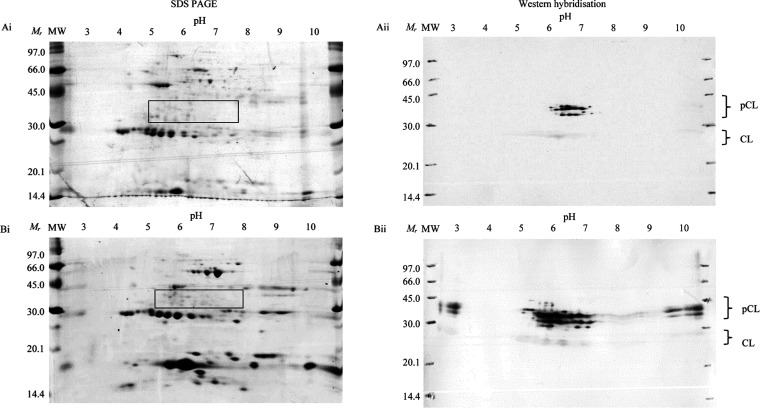
Representative 2-DE of *in vitro*-cultured live and dead adult *F. hepatica* excretory/secretory (ES) CL zymogen sub-proteomes and immunoreactivity against polyclonal anti-rFhΔpCL1 IgG. 25 μg ES products of live untreated (Ai) and dead (ethyl 4-aminobenzoate-terminated) (Bi) adult *F. hepatica* were analyzed by 2-DE. The area consisting of cathepsin L zymogens (≈30 to 38 kDa and 5.2–7.8 pI, boxed) were excised and analyzed by LC-MS^2^ (Table 2). 25 μg 2-DE-separated ES products of live untreated (Aii) and dead (ethyl 4-aminobenzoate-terminated) (Bii) adult *F. hepatica* were probed with anti-rFhΔpCL1 diluted to 1:5000. The greatest antigenicity was observed in protein spots separating at the same position as procathepsin L (pCL) and minor immunoreactivity of proteases (CL) in these native samples. Abbreviations: MW, Amersham Low Molecular Weight SDS Calibration Kit (*M*_r_).

*In vitro*-cultured live and dead (ethyl 4-aminobenzoate-terminated) adult fluke ES products were separated by 2-DE (Figure 4A,Bi), and LC-MS^2^ analysis of the target CL zymogen gel region was conducted (FhpCL; ≈30 to 38 kDa; 5.2–7.8 pI: Figure 4A,Bi, boxed). Three hits identified in the live sample were CLs (77.52% total average exponentially modified protein abundance index (emPAI)), whereas four of six hits in the dead sample were CLs (90.95% total average emPAI; excluding peptide samesets), as summarized in Table 2. Moreover, all CL hits in both samples indicated the recovery of peptides pertaining to pro-peptide, protease, and overlapping (inhibitor–protease) regions, indicating the presence of intact CL zymogens. An enolase and three hypothetical proteins sharing actin/-like protein signatures were also recovered in the dead fluke sample, likely due to their in-gel migration adjacent to CL zymogens (*F. hepatica* enolase ≈ 47 kDa, *F. hepatica* actin ≈ 41 kDa), as previously observed.^57^ In accordance with previous classification of cathepsin clades,^28^ the dead sample zymogen clade diversity contained CL1A (GenBank: AAP49831.1), CL2 (GenBank: ABQ95351.1), and CL5 (GenBank: AAF76330.1) clades compared to the live sample zymogens of the CL1 clade (CL1A, GenBank: AAB41670.2; CL1D, GenBank: ACJ12893.1). Thus, these findings demonstrate the feasibility of CL zymogen recovery from ES products derived from *in vitroF. hepatica* culture, in addition to increased diversity of CL clades in the dead *versus* live phenotype.

**Table 2:** LC-MS^2^ Identification of 2-DE-Separated *F. hepatica* CL Zymogen Sub-Proteomes^a^

	MS/MS ion search						highest-scoring GenBank hit			
				Sequence coverage						
ID	GenBank hit	MASCOT score (Av)	peptides matched (non-duplicate)	average percentage coverage (%)	collective residue coverage (aa)	exponentially modified protein abundance index (emPAI)	protein (length, aa)	organism	accession	*E*-value
live	**gi|116488416**^**a**^	**116.5 ± 58.5**	**4.0 ± 0.0**	**19.5 ± 1.5**	**57–81, 91–106, 116–124, 186–205, 231–253, 289–298, 308–324**	**0.365 ± 0.155**	**secreted cathepsin L1 (1–326)**	*F. hepatica*	AAB41670.2	**0.0**
	**gi|157862759**^**b**^	**101.0 ± 54.0**	**2.0 ± 0.0**	**11.5 ± 0.5**	**12–35, 70–78, 140–152, 185–207**	**0.280 ± 0.070**	**cathepsin L, partial (1–280)**	*F. gigantica*	ABV90502.1	**0.0**
	gi|211909240^b^	67.5 ± 20.5	3.0 ± 0.0	14.5 ± 1.5	58–81, 116–124, 186–198, 231–253, 289–298, 308–324	0.200 ± 0.010	cathepsin L1D (1–326)	*F. hepatica*	ACJ12893.1	0.0
dead	**gi|31558997**	**458.0 ± 260.0**	**10.5 ± 0.5**	**52.5 ± 6.5**	**42–81, 84–147, 186–205, 215–298**	**2.360 ± 1.830**	**cathepsin L (1–326)**	*F. hepatica*	AAP49831.1	**0.0 9E–180**
	**gi|41152540**	**384.5 ± 286.5**	**8.0 ± 1.0**	**52.5 ± 6.5**	**4–60, 99–118, 128–166, 202–211, 221–237**	**3.930 ± 3.510**	**cathepsin L protein (1–239)**	*F. hepatica*	AAR99519.1	**0.0**
	**gi|148575301**	**237.0 ± 152.0**	**10.5 ± 0.5**	**51.0 ± 2.0**	**50–81, 84–97, 106–115, 151–209, 215–302, 308–324**	**1.020 ± 0.840**	**secreted cathepsin L2 (1–326)**	*F. hepatica*	ABQ95351.1	**0.0**
	gi|190350155	153.5 ± 62.5	10.5 ± 0.5	33.0 ± 7.0		0.335 ± 0.025	enolase	*F. hepatica*	CAK47550.1	0.0
	gi|684403575	135.5 ± 37.5	14.0 ± 1.0	51.5 ± 7.5		0.440 ± 0.020	hypothetical protein T265_09499	*Opisthorchis viverrini*	XP 009173845.1	
	gi|684403578	135.5 ± 37.5	14.0 ± 1.0	46.0 ± 7.0		0.440 ± 0.020	hypothetical protein T265_09500	*O. viverrini*	XP 009173846.1	0.0
	gi|684415044	135.5 ± 37.5	7.5 ± 0.5	54.0 ± 11.0		0.440 ± 0.020	hypothetical protein T265_09500	*O. viverrini*	XP 009178086.1	3E-128
	**gi|8547325**	**126.0 ± 70**	**10.5 ± 0.5**	**33.5 ± 0.5**	**42–81,84–102, 116–124, 151–165, 186–198, 206–214, 254–266, 289–302**	**0.475 ± 0.295**	**Cathepsin L (1–326)**	*F. hepatica*	AAF76330.1	**0.0**

a CL zymogens in 2-DE-separated whole ES from untreated live and ethyl 4-aminobenzoate-terminated dead adult flukes (Figure 4A,Bi) were investigated by LC-MS. Protein hits are shown following identification against the GenBank database (v204) using an in-house Ma(Matrix Science) server, with consistent hits reported with average scores between duplicate sample submissions by two LC-MS^2^ methods (Agilent 6550 iFunnel Q-TOF (a) and Orbitrap Fusion Tribrid mass spectrometer (b)). Significant hits identified with an average score of 67 or greater (*P* < 0.05) are shown, including reliable error tolerance, reporting significant hits consistent between duplicate sample submissions, average abundance indices per hit (exponentially modified protein abundance index, emPAI), and showing protein family groupings in bold. Superscripts refer to consistent proteins identified as top hits from analyses by each LC-MS^2^ method.

Anti-rFhΔpCL1 polyclonal sera were probed *via* western hybridization against 2-DE-separated *in vitro*-cultured live and dead adult fluke ES products. IgG anti-ES recognition demonstrated an array of native endogenous procathepsin zymogens present in the live sample (Figure Figure 4Aii) and a larger range of immunoreactive procathepsin isoforms and protein spot abundance in the dead fluke sample (Figure 4Bii). Minor immunoreactivity of protein spots indicative of cathepsin proteases was also demonstrable at the antibody dilution used, which was reflected in relative reactivity between live and dead samples.

The presence or immunogenicity of intact CL zymogens from *in vitro*-cultured *F. hepatica* ES products has not been demonstrated until now, whereby the termination of active digesta expulsion (induced by ethyl 4-aminobenzoate treatment) caused detectable differences in ES profiles, including increased CL zymogen abundance (Figure 4; Table 2). When considering the LC-MS^2^ data alongside the western hybridizations, these findings correlate with the Morphew et al.^57^ study that demonstrated a reduction of mature CLs in dead worms when only investigating the mature proteins, suggesting protein abundances in death shift to fewer active mature CLs^57^ and more zymogen CLs (this study). Furthermore, these findings demonstrated multi-clade epitope homogeneity based on the diverse proteins, indicating anti-rFhΔpCL1 polyclonal IgG binding. However, unlike in the elucidation of the recombinant activated rFhpCL1_WT_-anti-rFhΔpCL1 profile (Figure 2), the present *F. hepatica* ES-anti-rFhΔpCL1 recognition profile (Figure 4) cannot confirm the involvement of isolated regional-specific epitopes or multiregion spanning conformational epitopes involved in immunorecognition.

### *In Silico* Procathepsin L Immunogenicity Predictions

The antigenicity of inhibitor- and protease-specific synthetic peptides have previously been tested, identifying diagnostically valuable CL protease-specific peptides^16,34^ and an immunoprotective CL inhibitor-specific peptide.^43^ However, following the protein recovery, identification, and demonstrable antigenicity of FhpCL zymogens from *in vitro* culture and recombinant protein fractions in this study, we sought to determine the underlying immunogenic peptides using the Kolaskar and Tongaonkar method^49^ to predict B cell-targeted epitopes.

As derived from our LC-MS^2^ data, *F. hepatica* CL zymogen protein sequences consisting of at least inhibitor peptide and protease regions were selected for analysis (1–310/326: (signal peptide−) inhibitor peptide–mature protease sequences), including eight hits (GenBank: ADP09371.1, 2O6X_A, AAB41670.2, CCA61803.1, AAP49831.1, ACJ12893.1, ABQ95351.1, AAF76330.1). Antigenic peptides of 7–28-mer were predicted in all sequences, with an average of 12.63 antigenic peptide determinants per sequence (Supporting Information: Supporting Figure S4). The fewest peptides (11 peptides) were predicted in 2O6X_A (CL1A, NB: signal sequence absent) and ABQ95351.1 (CL2), whereas ACJ12893.1 (CL1D) and AAF76330.1 (CL5) had the most (14 peptides) predicted determinants. Per sequence, peptides scoring above the average protein antigenicity were similarly located between all sequences, and the highest-scoring antigenic peptides (>1.1 average antigenic propensity) were present at the N-terminal (4–15 aa), mid-sequence (152–163 aa), and C-terminal (208–235; 283–289; and 311–321 aa). Antigenicity within zymogen-specific regions (1–108 aa) of these sequences was associated with 2–4 peptides overall between all eight sequences, and a further 7–11 peptides were also predicted in the protease-specific regions; however, a peptide predicted in ABQ95351.1 (CL2) overlapped both zymogen inhibitor- and protease-specific residues (90–110 aa).

The present immunogenic peptide predictions pertaining to all three protein regions of intact zymogen CLs are partially in keeping with the established use of intact protein and peptide-based diagnostics, which principally derive diagnostic efficacy from the mature protease region.^14,16^ Interestingly, when considering the absence of signal and inhibitor region-specific peptide immunoreactivity in the ES-anti-rFhΔpCL1 IgG recognition profiles (Figure 4A,Bii), this supports the interpretation of conformational-dependence for FhCL zymogen immunogenicity. However, the contributions of the inhibitor peptide toward anti-rFhΔpCL1 IgG reactivity when considering intact and cleaved zymogen antigen fractions (Figures 1–2B) is strongly supportive of the inhibitor peptide immunodominance, which could be plausible due to its reported conformational plasticity during autocatalysis.^17^ Hypothetically, however, region-specific immunogenicity could tie in with the naturally staggered release of these cleaved antigenic peptides as tactical decoys for immune evasion, which has been suggested for signal peptides in other disease models.^38,42,55^

### Detection of *In Vivo* Anti-FhpCL IgG and *Ex Vivo* FhpCL Fecal Antigen Capture

We have shown that the ES proteomic profiles of *in vitro*-cultured adult *F. hepatica* are demonstrably changed between live and dead flukes (Figure 4), and other studies have identified significant anthelmintic-induced changes between unexposed, TCBZ-exposed, and TCBZ-terminated fluke ES proteomes.^56,57^ However, the influence of TCBZ exposure and termination on the FhpCL subproteome, particularly for the immuno-proteomic comparison of TCBZ-S and TCBZ-R *F. hepatica* strains, is yet to be examined. Following the findings from our *in vitro*, *ex vivo*, and *in silico* FhpCL immuno-proteomic evaluations, we therefore sought to assess *in vivo* dynamics of endogenous FhpCL antigen exposure, release, and immunogenicity during *F. hepatica* experimental infections and TCBZ administration in livestock hosts. Moreover, we conceived to assess the differences in these phenotypes between TCBZ-S and TCBZ-R parasite infections and further identify the capacity for flukicide efficacy determination using two platforms, including serum IgG detection and fecal antigen capture.

**Figure 5:**
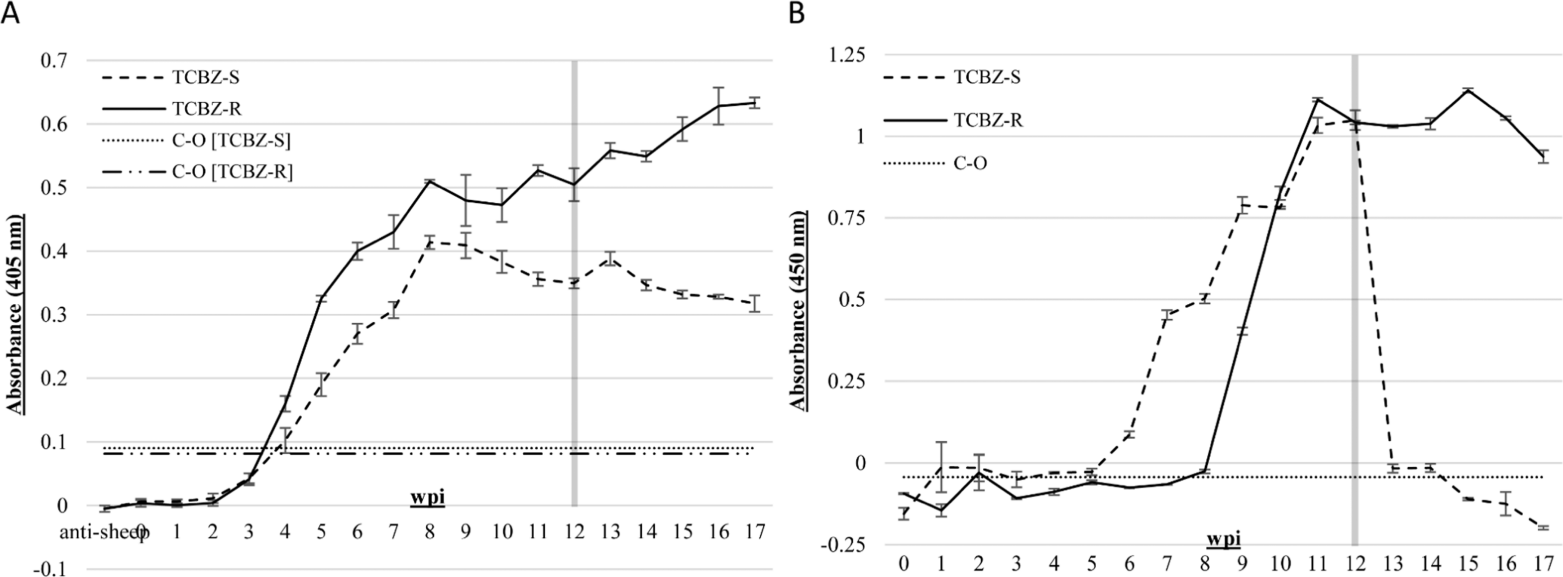
Validation of *F. hepatica* procathepsin L-based ELISA platforms for the comparison of antigen immunogenicity and capture during infection with TCBZ-S or TCBZ-R *F. hepatica* strains. Adjusted average ODs were calculated from two duplicate ELISA tests for both serum or fecal antigen capture ELISA platforms. (A) FhΔpCL1 Ag-ELISA was validated for serum antibody detection, whereby rFhΔpCL1 [0.5 μg/mL] was detected by experimental infection sera (1:750, *n* = 3 sheep, one parasite strain each) from 0–17 weeks post infection (wpi) with TCBZ-S (Aberystwyth, Italian, Miskin: dashed line) or TCBZ-R (Kilmarnock, Penrith, Stornoway: solid line) *F. hepatica* and following clinical administration of TCBZ at 12 wpi. Positive OD values for each sera group were considered when exceeding the cutoff (C–O), shown as one standard deviation above the negative Ag (BSA) OD score (dot line, TCBZ-S: 0.0901; dot-dash line, TCBZ-R: 0.0815). (B) Anti-rFhΔpCL1 IgG sandwich ELISA was validated for *F. hepatica* fecal antigen capture and identification of treatment success using anti-rFhΔpCL1 polyclonal IgG for capture and detection. Sheep fecal samples pooled from experimental infection fecal samples (*n* = 2 sheep, one parasite strain each) from 0–17 wpi, including TCBZ-S: Aberystwyth or Italian strains, or TCBZ-R: Kilmarnock or Stornoway strains. Positive OD values were considered when exceeding the −0.04329 OD cutoff (C–O; dot line), shown as one standard deviation above the highest average OD value measured for uninfected sheep samples. Error bars are one standard deviation above and below average ODs, and the shaded line indicates the time point of TCBZ administration.

To detect *in vivo* exposure and immunogenicity of FhpCL proteins, we used a direct ELISA format to test for rFhΔpCL1-binding IgG from experimentally infected sheep carrying TCBZ-S/-R *F. hepatica* isolates. Sheep serum samples pooled from experimental infections with strains of known TCBZ susceptibility (TCBZ-S: Aberystwyth, Italian, Miskin; or TCBZ-R: Kilmarnock, Penrith Stornoway) were tested, with weekly samples between 0–17 wpi and with TCBZ administration at 12 wpi. Based on IgG detection using a pNPP-AP-conjugated secondary antibody system, average OD measures were calculated from ELISAs conducted on two occasions and by subtracting the average OD of duplicate control (BSA, nonspecific antigen coating) wells from the average OD of duplicate test (rFhΔpCL1 antigen coating) wells. Serum positivity against the intact rFhΔpCL1 zymogen was determined after 4 and 5 wpi with TCBZ-R and TCBZ-S strains, respectively, followed by a shared peak in IgG binding between fluke phenotypes at 8 wpi (Figure 5A). Thereon, a steady increase in TCBZ-R-infected sample OD values was shown, whereas OD values of TCBZ-S-infected samples fell steadily until 17 wpi, with no significant decrease in antibody detection after 12 wpi in either phenotype (Figure 5A). Thus, FhpCL serum reactivity against rFhΔpCL1 was confirmed from both TCBZ-S/-R fluke infection phenotypes, and the continued positivity following TCBZ treatment in both sera groups was to be expected, given the long half-life of circulating IgG. However, despite the expected immunogenic potency of the signal/inhibitor peptide epitopes, the reactive IgG populations likely contain antibodies toward epitopes of the zymogen, protease, or both, which invites further differentiation of the FhpCL epitope-specificity of sera and zymogen antigen exposure.

Following the confirmed reactivity of TCBZ-S/-R *F. hepatica* infection sera to the rFhΔpCL1 antigen and to confirm *in vivo* FhpCL zymogen exposure, fecal antigen capture was used to detect endogenous excreted procathepsin zymogens, which was conducted using polyclonal anti-rFhΔpCL1 IgG(-biotin) in a sandwich ELISA. Sheep fecal samples pooled from experimental infections with TCBZ-S (Aberystwyth, Italian) or TCBZ-R (Kilmarnock, Stornoway) *F. hepatica* strains were tested, including all weekly intervals (0–17 wpi) and TCBZ administration at 12 wpi. Using coating anti-rFhΔpCL1 IgG and detection using the avidin–peroxidase system with anti-rFhΔpCL1 IgG-biotin to capture fecal antigens, average OD measures were calculated from ELISAs conducted on two occasions and by subtracting the average OD of duplicate control (nonimmunized rabbit IgG coating antibody) wells from the average OD of duplicate test (anti-rFhΔpCL1 rabbit IgG coating antibody) wells. OD data demonstrated positive values appearing from 4 wpi in the feces of TCBZ-S-infected sheep, climbing until 12 wpi, whereupon TCBZ administration induced a significant drop in OD (1 week post-treatment, 12–13 wpi: 97.36% OD reduction), leading to negative scores by 15 wpi (Figure 5B). Conversely, TCBZ-R-infected samples were positive from 8 wpi, whereupon OD scores increased sharply until 11 wpi, peaked at 15 wpi, then decreased at 16 and 17 wpi (Figure 5B). These data support the differential secretion patterns of FhpCL antigens detected by anti-rFhΔpCL1 IgG between TCBZ-S and TCBZ-R infection groups, including the first detection in feces during new infections and the evident TCBZ-induced termination of FhpCL production and detection in TCBZ-S fluke infections. Since current anthelmintic efficacy testing of parasite susceptibility requires the quantified reduction of 95% in fecal egg count or coproantigen (Bio-X Diagnostics, Belgium) levels by 2 weeks post-treatment, these findings also indicate the potential for faster diagnosis of anthelmintic efficacy.

### Determination of Anti-/rFhΔpCL1 Species Specificity

**Figure 6:**
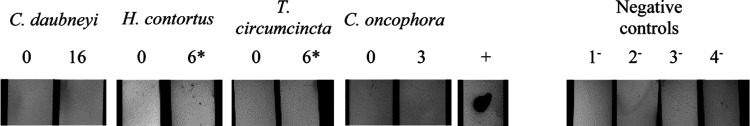
Dot blot analysis of IgG immunoreactivity of helminth-infected livestock serum against rFhΔpCL1. rFhΔpCL1 (0.01 μg/dot) was probed with pooled whole serum diluted to 1:700 (*n* = 2 sheep, with either *C. daubneyi*, *H. contortus*, or *O. circumcinta*), 1:100 (*n* = 2 cattle, with *C. oncophora* infection), or 1:5000 (*n* = 2 rabbits immunized with anti-rFhΔpCL1), and IgG binding was detected using anti-sheep, anti-cattle, or anti-rabbit IgG at 1:30,000 per appropriate sample and the BCIP-NBT system until a precipitant appearance in the positive control. Negative controls include: 1^–^, pre-[rFhΔpCL1] immunization; 2^–^, anti-bovine (2° antibody only); 3^–^, anti-sheep (2° antibody only); and 4^–^, anti-rabbit (2° antibody only). The asterisk (*) indicates these sera were collected at day 39 (between 5–6 wpi). Abbreviations: +, positive control; −, negative control; and wpi, week(s) post infection.

The specificity of the anti-rFhΔpCL1 IgG/-biotin sandwich ELISA was assessed to ensure the test correctly identified samples with known negativity for *F. hepatica* infection. As such, fecal samples from livestock hosts infected with non-*F. hepatica* helminths were used, including *C. daubneyi* (*n* = 2 cattle, 12 wpi), *H. contortus* (*n* = 2 sheep, 6 wpi), or *T. circumcincta* (*n* = 2 sheep, 6 wpi), and data were collated from two ELISA plates conducted on separate occasions. No average test OD values exceeded the control cutoffs for sheep or cattle, but due to high background levels, further OD values for each test group were re-calculated by subtracting the lowest test OD from anti-rFhΔpCL1 IgG-coated wells, which remained below the cutoff and was thus considered to be negative. Further assessments of cross-reactivity were conducted using dot blots to determine the reactivity of livestock sera infected with non-*F. hepatica* helminth parasites against rFhΔpCL1. Sera samples were pooled from two sheep or cattle infected with U.K.-endemic livestock helminths, including *C. daubneyi* (*n* = 2 sheep, 0 and 16 wpi), *H. contortus* (*n* = 2 sheep, 0 and 6 wpi (day 39)), *T. circumcincta* (*n* = 2 sheep, 0 and 6 wpi (day 39)), and *C. oncophora* (*n* = 2 cattle, 0 and 3 wpi). Based on these data, there was no visible immunoreactivity of any sera against rFhΔpCL1 to indicate cross-reactivity and/or equivalent species-specific antigen exposures *in vivo* (Figure 6). Overall, therefore, these findings support the diagnostic specificity of rFhΔpCL1 and anti-rFhΔpCL1 IgG tools for the determination of *F. hepatica* infection and negativity of infection samples by other common coexisting livestock parasitic helminths.

## Conclusions

Cathepsin L (CL) proteases have been a major molecular focus of *F. hepatica* research for many years. However, using recombinant and native procathepsins and counterpart polyclonal antibodies to a recombinant FhpCL1A, we have demonstrated multiple highly antigenic and conformationally dependent epitopes of diagnostic potential for fasciolosis and anthelmintic efficacy evaluation.

The identification and comparative study of proteomic differences between *F. hepatica* of live and dead groups, untreated and TCBZ-exposed groups, and TCBZ-S and TCBZ-R strains have identified numerous molecular diagnostic and vaccine candidates. FhpCL procathepsin zymogens had previously remained as a large collection of unexploited antigens, but data here have definitively confirmed the highly immunodominant zymogen segment of the well-known cathepsin L proteins and further show encouraging potential as diagnostic antigens. Binding patterns by anti-rFhΔpCL1 IgG toward recombinant and native CL zymogens here show immunoreactivity is sustained within recombinant proteins in the CL1A clade and between multiple native adult-specific pro-enzymes of clades CL1, CL2, and CL5. Furthermore, mature proteases of either recombinant or native samples did not elicit analogous recognition akin to zymogen peptide-associated fractions, supporting zymogen-specific epitope immunodominance.

Overall, we identified multiple conserved, immunodominant epitopes of *in vitroF. hepatica* procathepsin L zymogens and showed FhpCL antigens are exposed to the host immune system *in vivo* and moreover are secreted as coproantigens, which can be used to indicate treatment efficacy in experimental TCBZ-S/-R infections. The standardization of these FhpCL-based test platforms with natural samples will allow penside/point-of-care applications to support diagnosis and anthelmintic efficacy testing of *F. hepatica* infections.

## Supporting Information Available

Western hybridization images (Figure S1); sequence alignment of distinct LC-MS^2^ hits (Figure S2); amido black NCM transfer confirmation (Figure S3); antigenicity predictions and sequence alignment of FhpCLs (Figure S4); nomenclature of mass spectrometry samples data submission (Table S1); comprehensive data from all LC-MS^2^ analyses (Table S2) (PDF)

## Abbreviations

1-/2-DE: 1-/2-dimensional electrophoresis
BCIP-NBT: 5-bromo-4-chloro-3-indolyl-phosphate-nitro blue tetrazolium
ELISA: enzyme-linked immunosorbent assay
ES: excretory/secretory
LC-MS^2^: liquid chromatography-tandem mass spectrometry
(r)FhpCL: (recombinant) *Fasciola hepatica* procathepsin L
SDS PAGE: sodium dodecyl sulfate polyacrylamide gel electrophoresis
TCBZ-S/-R: triclabendazole-susceptible/-resistant
wpi: week(s) post infection
