# Supplementary material for: Fasciola hepatica Cathepsin L Zymogens: Immuno-Proteomic Evidence for Highly Immunogenic Zymogen-Specific Conformational Epitopes to Support Diagnostics Development
Source: J Proteome Res. 2022 Jul 18;21(8):1997–2010. doi: 10.1021/acs.jproteome.2c00299 (PMC9361350; doi:10.1021/acs.jproteome.2c00299)
Supplement: Supplementary file 1 — pr2c00299_si_001.pdf [file pr2c00299_si_001.pdf]

Fasciola hepatica cathepsin L zymogens: immuno-proteomic evidence for highly immunogenic zymogen-specific conformational epitopes to support diagnostics development

Clare F. Collett[1][†], Helen C. Phillips[1], Maggie Fisher[2], Sian Smith[2], Caroline Fenn[2], Phil Goodwin[3], Russell M. Morphew[1] and Peter M. Brophy[1]*

[1] Institute of Biological, Environmental and Rural Sciences, Aberystwyth University, Aberystwyth, SY23 3DA, UK. [2] Ridgeway Research Ltd., Park Farm Buildings, Park Lane, St. Briavels, Gloucestershire, GL15 6QX, UK. [3] Bio-Check UK, Spectrum House, Llys Edmund Prys, St. Asaph Business Park, St. Asaph, Denbighshire, LL17 0LJ, UK. † Present address: UK Health Security Agency, Salisbury, SP4 0JG, UK.

Supporting Information table of contents

Section	Page number
Cover page	**S-1**
Supplementary Figure S1	**S-2**
Supplementary Figure S2	**S-3–4**
Supplementary Figure S3	**S-5**
Supplementary Figure S4	**S6–7**
Supplementary Table S1	**S8–9**
Supplementary Table S2	**S10–14**

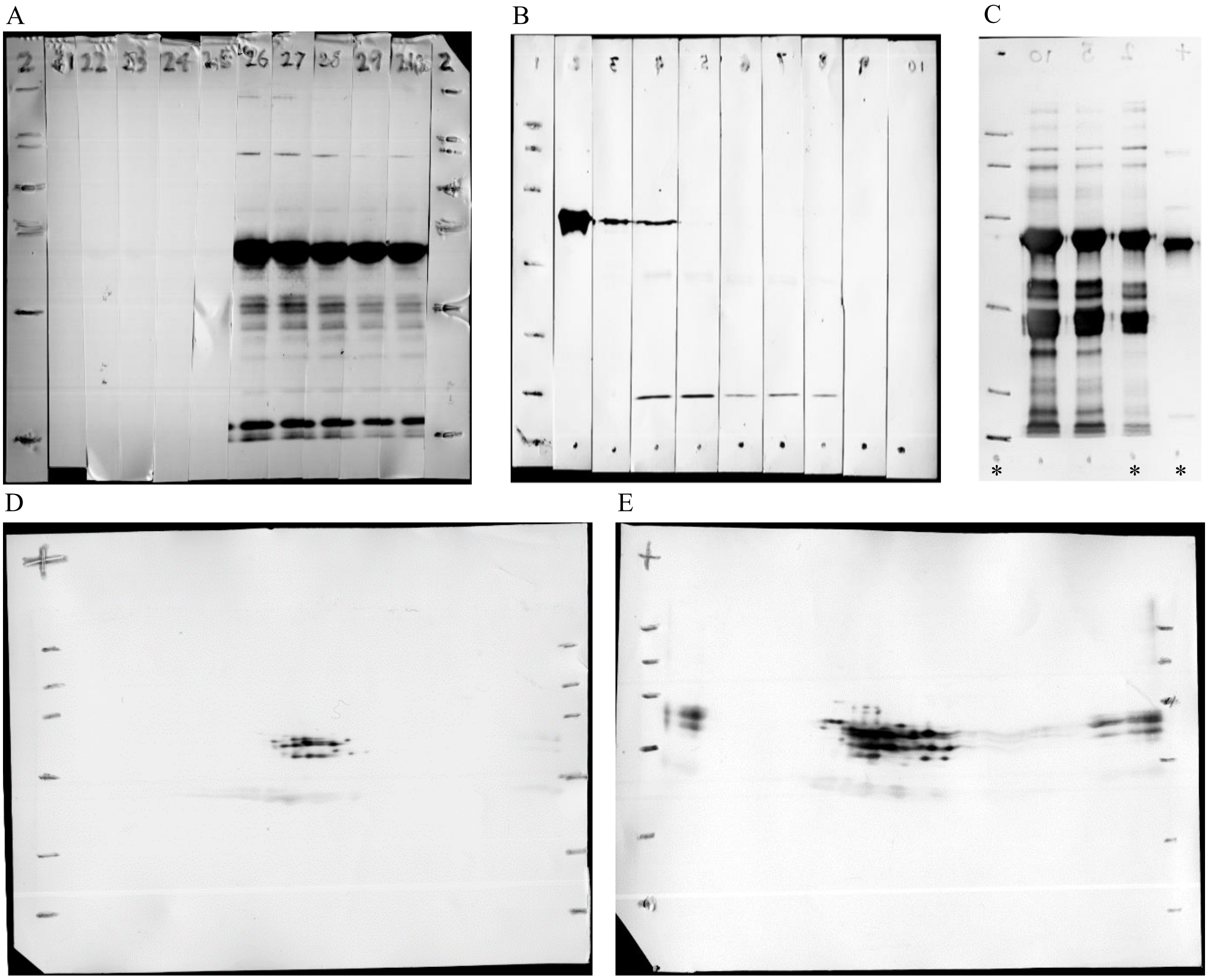

Supplementary Figure S1. Uncropped images of entire membranes from western hybridisation procedures. Images are shown in order of presentation in the article: including from the western hybridisations with anti-rFhΔpCL1 IgG against 1-DE-separated recombinant procathepsins (A–C; Figures 1–3B) and 2-DE-separated *in vitro*-derived ES products from live and (D; Figure 4Aii) and dead (E; Figure 4Bii) *F. hepatica*. (C) 10, 5 and 2 μg per lane of rFhpCL1 was probed, with greatest image clarity achieved at the lowest quantity (pre-selected lanes used in Figure 3B are depicted: *).

A

```
2O6X_A          ----------------NDDLWHQWKRMYNKEYNGADDQHRRNIWEKNVKHIQEHNLRHDL
ADP09371.1      MRLFILAVLTVGVLGSNDDLWHQWKRMYNKEYNGADDQHRRNIWEKNVKHIQEHNLRHDL
AAB41670.2      MRLFVLAVLTVGVLGSNDDLWHQWKRMYNKEYNGADDQHRRNIWEKNVKHIQEHNLRHDL
                                ********************************************

2O6X_A          GLVTYTLGLNQFTDMTFEEFKAKYLTEMSRASDILSHGVPYEANNRAVPDKIDWRESGYV
ADP09371.1      GLVTYTLGLNQFTDMTFEEFKAKYLTEMSRASDILSHGVPYEANNRAVPDKIDWRESGYV
AAB41670.2      GLVTYTLGLNQFTDMTFEEFKAKYLTEMSRASDILSHGVPYEANNRAVPDKIDWRESGYV
                ************************************************************

2O6X_A          TEVKDQGNCGSGWAFSTTGTMEGQYMKNERTSISFSEQQLVDCSRPWGNNGCGGGLMENA
ADP09371.1      TEVKDQGNCGSCWAFSTTGTMEGQYMKNERTSISFSEQQLVDCSRPWGNNGCGGGLMENA
AAB41670.2      TEVKDQGNCGSCWAFSTTGTMEGQYMKNERTSISFSEQQLVDCSRPWGNNGCGGGLMENA
                *********** ************************************************

2O6X_A          YQYLKQFGLETESSYPYTAVEGQCRYNKQLGVAKVTGFYTVHSGSEVELKNLVGAEGPAA
ADP09371.1      YEYLKQFGLETESSYPYRAVEGQCRYNKQLGVAKVTGYYTVHSGSEVELKNLVGAEGPAA
AAB41670.2      YQYLKQFGLETESSYPYTAVEGQCRYNKQLGVAKVTGFYTVHSGSEVELKNLVGAEGPAA
                *:*************** *******************:**********************

2O6X_A          VAVDVESDFMMYRSGIYQSQTCSPLRVNHAVLAVGYGTQGGTDYWIVKNSWGLSWGERGY
ADP09371.1      VAVDVESDFMMYSGGIYQSQTCSPLGLNHAVLAVGYGTQGGTDYWIVKNSWGLSWGERGY
AAB41670.2      VAVDVESDFMMYRSGIYQSQTCSPLRVNHAVLAVGYGTQGGTDYWIVKNSWGLSWGERGY
                ************ .*********** :*********************************

2O6X_A          IRMVRNRGNMCGIASLASLPMVARFP
ADP09371.1      IRMARNRGNMCGIASLASLLMVARFP
AAB41670.2      IRMVRNRGNMCGIASLASLPMVARFP
                ***.*************** ******
```

Percent identity matrix	ADP09371.1	AAB41670.2
2O6X_A	96.77	99.68
ADP09371.1		96.93

B

```
CCA61803.1      MRLFILAVLTVGVLGSNDDLWHQWKRMYNKEYNGDDDQHRRNIWEKNVKHIQEHNLRHDL
ADP09371.1      MRLFILAVLTVGVLGSNDDLWHQWKRMYNKEYNGADDQHRRNIWEKNVKHIQEHNLRHDL
AAR99519.1      ------------------------------------------------------------

CCA61803.1      GLVTYTLGLNQFTDMTFEEFKAKYLTEMSRASDILSHGVPYEANNRAVPDKIDWRESGYV
ADP09371.1      GLVTYTLGLNQFTDMTFEEFKAKYLTEMSRASDILSHGVPYEANNRAVPDKIDWRESGYV
AAR99519.1      ---------------------------MSRASDILSHGIPYEANNRAVPDKIDWRESGYV
                                           ***********:*********************
CCA61803.1      TEVKDQGNCGSCWAFSTTGTMEGQYMKNERTSISFSEQQLVDCSGPWGNNGCSGGLMENA
ADP09371.1      TEVKDQGNCGSCWAFSTTGTMEGQYMKNERTSISFSEQQLVDCSRPWGNNGCGGGLMENA
AAR99519.1      TGVKDQGNCGSCWAFSTTGTMEGQYMKNERTSISFSEQQLVDCSGPWGNNGCSGGLMENA
                * ****************************************** *******.*******

CCA61803.1      YQYLKQFGLETESSYPYTAVEGQCRYNKQLGVAKVTGYYTVHSGSEVELKNLVGAEGPAA
ADP09371.1      YEYLKQFGLETESSYPYRAVEGQCRYNKQLGVAKVTGYYTVHSGSEVELKNLVGAEGPAA
AAR99519.1      YQYLKQFGLETESSYPYTAVEGQCRYNRQLGVAKVTGYYTVHSGSEVELKNLVGSEGPAA
                *:*************** *********:**************************:*****


CCA61803.1      VAVDVESDFMMYSGGIYQSQTCSPLGLNHAVLAVGYGTQGGTDYWIVKNSWGSYWGERGY
ADP09371.1      VAVDVESDFMMYSGGIYQSQTCSPLGLNHAVLAVGYGTQGGTDYWIVKNSWGLSWGERGY
AAR99519.1      IAVDVESDFMMYRSGIYQSQTCLPFALNHAVLAVGYGTQGGTDYWIVKNSWGLSWGERGY
                :*********** .******** *:.**************************  ******

CCA61803.1      IRMARNRGNMCGIASLASLPMVARFP
ADP09371.1      IRMARNRGNMCGIASLASLLMVARFP
AAR99519.1      IRMARNRGNMCGIASLASLPMVARFP
                ******************* ******
```

Percent identity matrix	ADP09371.1	AAR99519.1
CCA61803.1	97.55	94.98
ADP09371.1		93.72

Supplementary Figure S2. Sequence alignment for recombinant *F. hepatica* procathepsin L proteins identified from LC-MS². The top two LC-MS² hits from rFhΔpCL1 (A) and

rFhpCL1 (B) were aligned against the highest scoring common BLAST hit to determine the protein familial clade identity as a CL1A, with corresponding percentage identity shown. (A) Residue differences between the top LC-MS^2 hits for rFhΔpCL1 are also highlighted in grey.

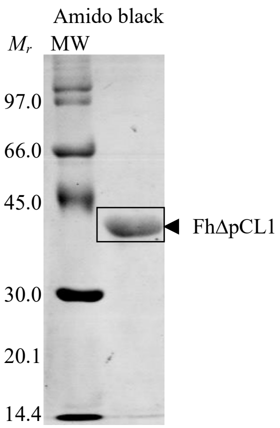

Supplementary Figure S3. Amido black confirmation of rFhΔpCL1 transfer to NCM. Electrophoretic transfer of the rFhΔpCL1 zymogen to NCM following 1-DE separation was confirmed using amido black staining (boxed: expected molecular weight, 36.9 kDa). Abbreviations: MW, Amersham Low Molecular Weight SDS Calibration Kit (Mr).

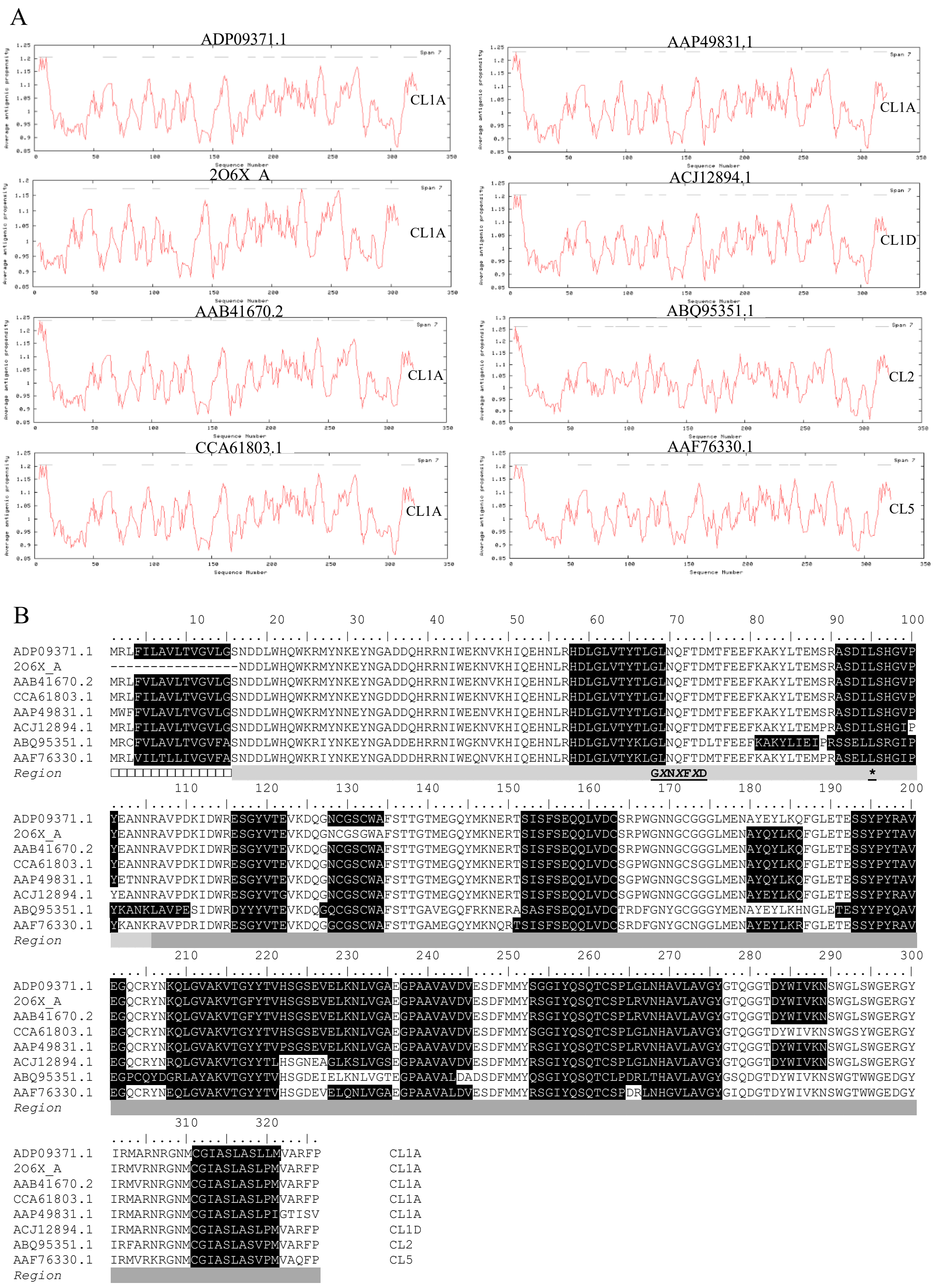

Supplementary Figure S4. Antigenicity profile predictions for *F. hepatica* procathepsin L protein sequences. FhpCL sequences of interest identified from recombinant and native LC-

MS^2 analyses in this study were analysed for putative B cell epitopes (A), identifying multiple antigenic residues and peptides that scored above average antigenicity scores per whole protein sequence. (B) 14 major sections of each sequence comprising antigenic determinants of interest are highlighted, with broadly conserved antigenic peptides across all CL protein sequences analysed and minor deviations in antigenicity at residue substitutions. Regions of interest are also highlighted, including the signal (pre-) peptide (1–15 aa: □), inhibitor (pro-) peptide (16–105 aa: light grey), and protease (106–326 aa: dark grey), the procathepsin autoactivation motif (G*X*N*X*F*X*D), and the Leu95Pro *in situ* residue substitution of the recombinant mutant procathepsin L (rFhΔpCL1).

Supplementary Table S1. Detailed nomenclature of mass spectrometry samples submitted using PRIDE to the ProteomeXchange Consortium (data identifier: PXD030293).[47] Instruments used for mass spectrometry are indicated per sample, including *1: Agilent 6550 iFunnel Q-TOF, or *2: Orbitrap Fusion.

File name	Data category	Replicate	LC-MS^2 instrument*
**160627-cc_2.dat**<br>**160627-cc_2.mgf**<br>**MutantCL_rep1-160627-cc_2.mzid.gz**	Raw<br>Peak<br>Result	1	1
**180815-Sample23.d.zip**<br>**180815-Sample23.mgf**<br>**MutantCL_rep2-180815-Sample23.mzid.gz**	Raw<br>Peak<br>Result	2	1
**180814-Sample19.d.zip**<br>**180814-Sample19.mgf**<br>**CLwt_37_rep1-180814-Sample19.mzid.gz**	Raw<br>Peak<br>Result	1	1
**180814-Sample21.d.zip**<br>**180814-Sample21.mgf**<br>**CLwt_37_rep2-180814-Sample21.mzid.gz**	Raw<br>Peak<br>Result	2	1
**180814-Sample20.d.zip**<br>**180814-Sample20.mgf**<br>**CLwt_35_rep1-180814-Sample20.mzid.gz**	Raw<br>Peak<br>Result	1	1
**180815-Sample22.d.zip**<br>**180815-Sample22.mgf**<br>**CLwt_35_rep2-180815-Sample22.mzid.gz**	Raw<br>Peak<br>Result	2	1
**180815-Sample26.d.zip**<br>**180815-Sample26.mgf**<br>**rFhpCL1-37-180815-Sample26.mzid.gz**	Raw<br>Peak<br>Result	1	1
**180815-Sample32.d.zip**<br>**180815-Sample32.mgf**<br>**rFhpCL1-37-180815-Sample32.mzid.gz**	Raw<br>Peak<br>Result	2	1
**180815-Sample27.d.zip**<br>**180815-Sample27.mgf**<br>**rFhpCL1-32-180815-Sample27.mzid.gz**	Raw<br>Peak<br>Result	1	1
**180815-Sample33.d.zip**<br>**180815-Sample33.mgf**<br>**rFhpCL1-32-180815-Sample33.mzid.gz**	Raw<br>Peak<br>Result	2	1
**180815-Sample28.d.zip**<br>**180815-Sample28.mgf**<br>**rFhpCL1-28-180815-Sample28.mzid.gz**	Raw<br>Peak<br>Result	1	1
**180815-Sample34.d.zip**<br>**180815-Sample34.mgf**<br>**rFhpCL1-28-180815-Sample34.mzid.gz**	Raw<br>Peak<br>Result	2	1
**180815-Sample29.d.zip**<br>**180815-Sample29.mgf**<br>**rFhpCL1-24-180815-Sample29.mzid.gz**	Raw<br>Peak<br>Result	1	1
**180815-Sample35.d.zip**<br>**180815-Sample35.mgf**<br>**rFhpCL1-24-180815-Sample35.mzid.gz**	Raw<br>Peak<br>Result	2	1
**180815-Sample30.d.zip**<br>**180815-Sample30.mgf**<br>**rFhpCL1-18-180815-Sample30.mzid.gz**	Raw<br>Peak<br>Result	1	1
**180815-Sample36.d.zip**<br>**180815-Sample36.mgf**<br>**rFhpCL1-18-180815-Sample36.mzid.gz**	Raw<br>Peak<br>Result	2	1

**180815-Sample31.d.zip**<br>**180815-Sample31.mgf**<br>**rFhpCL1-14-180815-Sample31.mzid.gz**	Raw<br>Peak<br>Result	1	1
**180815-Sample37.d.zip**<br>**180815-Sample37.mgf**<br>**rFhpCL1-14-180815-Sample37.mzid.gz**	Raw<br>Peak<br>Result	2	1
**Live_ES_rep1-170220-Sample1.mzid.gz**<br>**170220-Sample1.mgf**<br>**Live_ES_rep1-170220-Sample1.mzid.gz**	Raw<br>Peak<br>Result	1	1
**1_31.raw**<br>**1_31.mgf**<br>**Live_ES_rep2-190322-O-Clare-2D-MGF-1_31.mzid.gz**	Raw<br>Peak<br>Result	2	2
**170220-Sample2.dat**<br>**170220-Sample2.mgf**<br>**Dead_ES_rep1-170220-Sample2.mzid.gz**	Raw<br>Peak<br>Result	1	1
**1_32.raw**<br>**1_32.mgf**<br>**Dead_ES_rep2-190322-O-Clare-2D-MGF-1_32.mzid.gz**	Raw<br>Peak<br>Result	2	2
**180815-Sample51.d.zip**<br>**180815-Sample51.mgf**<br>**ESI-QUAD-TOF_Neg_Control-180815-Sample51.mzid.gz**	Raw<br>Peak<br>Result	n/a	1
**1_c1.raw**<br>**1_c1.mgf**<br>**ESI_TRAP_Neg_Control-1_c1.mzid.gz**	Raw<br>Peak<br>Result	n/a	2

Supplementary Table S2. Comprehensive LC-MS^2 identifications of 1-DE-separated recombinant *Fasciola hepatica* CL zymogens. LC-MS^2 analyses of 12.5% SDS PAGE-separated recombinant mutant (rFhΔpCL1) and wild type (rFhpCL1$_{WT}$) procathepsin L (Ireland) and a second recombinant procathepsin L (Spain) (Figures 2b–4b) were conducted, and protein hits were identified against the GenBank database (v204) using an in-house MASCOT (Matrix Science) server. Hits were included where consistent between duplicates with an average score of 67 or greater ($P < 0.05$) including reliable error tolerance, and both top hits (**bolded**, Table 1) and all other hits (plain text), including hits derived from peptide samesets, subsets and intersections, are shown. For the latter hits, the numbers of peptides matched were calculated from those of the original hits. The asterisk (*) indicates a protein hit identified across both replicates for rFhpCL1 sample 5, but absent from all other samples including the negative controls, where 144 peptide samesets, subsets and intersections were associated but excluded from this summary.

rFhpCL1	Approximate molecular weight (sample number)	GenBank hit	MASCOT score (Av)	Peptides matched (non-duplicate)	Sequence coverage (%)	Protein	Organism	Accession	E-value
rFhΔpCL1	37	**gi|163310848**	**1677.0±1078.0**	**71.5±37.5**	**73.0±10.0**	**Chain A, Crystal Structure Of Procathepsin L1**	***Fasciola hepatica***	**2O6X_A**	**0.0**
		gi|116488416	1582.0±1076.0	71.5±37.5	62.0±9.0	Secreted cathepsin L 1	*F. hepatica*	AAB41670.2	0.0
		gi|31558997	1419.0±1059.0	71.5±37.5	40.0±5.0	Cathepsin L	*F. hepatica*	AAP49831.1	0.0
		gi|377823949	1309.0±899.0	71.5±37.5	34.0±0.0	Cathepsin L1	*F. gigantica*	AFB77219.1	0.0
		gi|20136379	1233.5±862.5	71.5±37.5	44.5±2.5	Cathepsin L, partial	*F. hepatica*	AAM11647.1	0.0
		gi|157862759	1056.5±898.5	71.5±37.5	23.0±3.0	Cathepsin L, partial	*F. gigantica*	ABV90502.1	0.0
		gi|74765984	864.5±453.5	71.5±37.5	44.0±6.0	Cathepsin L-like proteinase	*F. hepatica*	Q24940.1	0.0
		gi|7271889	805.5±490.5	71.5±37.5	30.5±3.5	Cathepsin L	*F. gigantica*	AAF44675.1	0.0
		gi|4574304	801.0±491.0	71.5±37.5	30.5±3.5	Cathepsin	*F. gigantica*	AAD23996.1	0.0
		gi|379991182	618.5±300.5	71.5±37.5	35.0±4.0	Cathepsin protein CatL1-MM3p, partial	*F. hepatica*	CCA61803.1	0.0
		gi|13774082	612.0±297.0	71.5±37.5	34.5±2.5	Cathepsin L-like	*F. hepatica*	AAK38169.1	0.0
		gi|41152538	612.0±297.0	71.5±37.5	37.0±5.0	Cathepsin L protein	*F. hepatica*	AAR99518.1	0.0
		gi|21263041	612.0±297.0	71.5±37.5	27.0±0.0	Cathepsin L2	*F. gigantica*	AAM44832.1	0.0
		gi|211909242	501.5±290.5	71.5±37.5	24.5±3.5	Cathepsin L1D	*F. hepatica*	ACJ12894.1	0.0
		gi|10798511	497.5±294.5	71.5±37.5	23.5±1.5	Cathepsin L1	*F. hepatica*	CAC12806.1	0.0
		gi|211909240	497.5±294.5	71.5±37.5	24.5±3.5	Cathepsin L1D	*F. hepatica*	ACJ12893.1	0.0
		gi|545734	493.0±192.0	71.5±37.5	21.0±5.0	Cysteine protease	*Fasciola sp.*	AAB30089.1	0.0
		**gi|310751866**	**441.5±281.5**	**30.5±17.5**	**39.0±8.0**	**Cathepsin L-like proteinase**	***F. hepatica***	**ADP09371.1**	**0.0**
		gi|7271895	383.5±187.5	71.5±37.5	20.0±9.0	Cathepsin L, partial	*F. gigantica*	AAF44678.1	1E-164
		gi|7271891	364.0±295.0	71.5±37.5	16.5±1.5	Cathepsin L	*F. gigantica*	AAF44676.1	0.0
		gi|107921814	355.5±298.5	71.5±37.5	10.5±0.5	Cathepsin L4	*F. hepatica*	ABF85682.1	2E-172
		gi|195729975	355.5±298.5	71.5±37.5	8.5±1.5	Cathepsin L1	*Fascioloides magna*	ACG50798.1	0.0
		gi|167427529	355.5±298.5	71.5±37.5	7.5±0.5	Cathepsin L4, partial	*F. hepatica*	ABZ80401.1	0.0
		gi|38045864	355.5±298.5	71.5±37.5	7.0±0.0	Cathepsin L	*F. gigantica*	AAR08900.1	0.0
		gi|146147376	302.0±1.0	71.5±37.5	25.5±5.5	Cathepsin	*F. gigantica*	ABQ01982.1	0.0
		gi|50403821	301.5±2.5	71.5±37.5	24.5±4.5	Cathepsin L1 proteinase	*F. hepatica*	AAT76664.1	0.0
		gi|41152540	297.5±1.5	71.5±37.5	38.0±5.0	Cathepsin L protein	*F. hepatica*	AAR99519.1	9E-180
		gi|108735840	193.0±2.0	71.5±37.5	22.0±0.0	Cathepsin L2	*F. hepatica*	ABG00259.1	7E-167
		gi|19909509	154.0±1.0	71.5±37.5	21.5±4.5	Cathepsin L	*F. gigantica*	BAB86959.1	0.0
		gi|452266	154.0±1.0	71.5±37.5	23.0±11.0	Cathepsin L-like protease	*F. hepatica*	CAA80450.1	8E-124
		gi|8547325	90.5±21.5	19.5±14.5	8.0±6.0	Cathepsin L	*F. hepatica*	AAF76330.1	0.0
rFhpCL1$_{WT}$	37	**gi|116488416**	**132.5±56.5**	**17.0±4.0**	**38.5±1.5**	**Secreted cathepsin L 1**	***F. hepatica***	**AAB41670.2**	**0.0**
		gi|163310848	132.5±56.5	17.0±4.0	40.5±1.5	Chain A, Crystal Structure Of Procathepsin L1	*F. hepatica*	2O6X_A	0.0
		gi|74765984	130.5±54.5	17.0±4.0	33.0±4.0	Cathepsin L-like proteinase	*F. hepatica*	Q24940.1	0.0
		gi|4574304	107.0±42.0	17.0±4.0	20.0±0.0	Cathepsin	*F. gigantica*	AAD23996.1	0.0
		gi|545734	107.0±42.0	17.0±4.0	21.5±1.5	Cysteine protease	*Fasciola sp.*	AAB30089.1	0.0
		gi|7271889	107.0±42.0	17.0±4.0	21.5±1.5	Cathepsin L	*F. gigantica*	AAF44675.1	0.0
		gi|31558997	102.0±26.0	17.0±4.0	23.5±1.5	Cathepsin L	*F. hepatica*	AAP49831.1	0.0

		gi|7271895	96.5±35.5	17.0±4.0	19.0 ±2.0	Cathepsin L, partial	*F. gigantica*	AAF44678.1	1E-164
		gi|379991182	83.0±39.0	17.0±4.0	22.5±3.5	Cathepsin protein CatL1-MM3p, partial	*F. hepatica*	CCA61803.1	0.0
		gi|20136379	79.0±22.0	17.0±4.0	22.0±4.0	Cathepsin L, partial	*F. hepatica*	AAM11647.1	0.0
		gi|13774082	77.0±33.0	17.0±4.0	22.0±4.0	Cathepsin L-like	*F. hepatica*	AAK38169.1	0.0
		gi|41152538	77.0±33.0	17.0±4.0	20.5±3.5	Cathepsin L protein	*F. hepatica*	AAR99518.1	0.0
		gi|41152540	77.0±33.0	17.0±4.0	26.5±3.5	Cathepsin L protein	*F. hepatica*	AAR99519.1	9E-180
		gi|50403821	77.0±33.0	17.0±4.0	19.5±2.5	Cathepsin L1 proteinase	*F. hepatica*	AAT76664.1	0.0
		gi|21263041	77.0±33.0	17.0±4.0	15.0±1.0	Cathepsin L2	*F. gigantica*	AAM44832.1	0.0
		gi|146147376	77.0±33.0	17.0±4.0	15.0±1.0	Cathepsin	*F. gigantica*	ABQ01982.1	0.0
		gi|377823949	77.0±33.0	17.0±4.0	15.0±1.0	Cathepsin L1	*F. gigantica*	AFB77219.1	0.0
rFhpCL1$_{WT}$	35	**gi|116488416**	**117.0±51.0**	**13.5±3.5**	**41.0±4.0**	**Secreted cathepsin L 1**	***F. hepatica***	**AAB41670.2**	**0.0**
		gi|163310848	117.0±51.0	13.5±3.5	43.0±4.0	Chain A, Crystal Structure Of Procathepsin L1	*F. hepatica*	2O6X_A	0.0
		gi|74765984	116.5±51.5	13.5±3.5	35.5±6.5	Cathepsin L-like proteinase	*F. hepatica*	Q24940.1	0.0
		gi|4574304	104.0±45.0	13.5±3.5	21.5±3.5	Cathepsin	*F. gigantica*	AAD23996.1	0.0
		gi|545734	104.0±45.0	13.5±3.5	21.5±1.5	Cysteine protease	*Fasciola sp.*	AAB30089.1	0.0
		gi|7271889	104.0±45.0	13.5±3.5	24.0±4.0	Cathepsin L	*F. gigantica*	AAF44675.1	0.0
		gi|31558997	94.5±29.5	13.5±3.5	26.0±4.0	Cathepsin L	*F. hepatica*	AAP49831.1	0.0
		gi|20136379	86.0±33.0	13.5±3.5	24.5±6.5	Cathepsin L, partial	*F. hepatica*	AAM11647.1	0.0
		gi|7271895	79.0±20.0	13.5±3.5	21.0±3.0	Cathepsin L, partial	*F. gigantica*	AAF44678.1	1E-164
rFhpCL1	37 (1)	**gi|379991182**	**90.5±9.5**	**10.5±3.5**	**30.0±4.0**	**Cathepsin protein CatL1-MM3p, partial**	***F. hepatica***	**CCA61803.1**	**0.0**
		gi|74765984	87.0±13.0	10.5±3.5	27.0±4.0	Cathepsin L-like proteinase	*F. hepatica*	Q24940.1	0.0
		gi|116488416	87.0±13.0	10.5±3.5	24.0±4.0	Cathepsin L-like	*F. hepatica*	AAK38169.1	0.0
		gi|163310848	87.0±13.0	10.5±3.5	22.5±3.5	Chain A, Crystal Structure Of Procathepsin L1	*F. hepatica*	2O6X_A	0.0
		gi|13774082	86.5±12.5	10.5±3.5	22.5±3.5	Secreted cathepsin L 1	*F. hepatica*	AAB41670.2	0.0
		gi|41152538	86.5±12.5	10.5±3.5	22.5±3.5	Cathepsin L protein	*F. hepatica*	AAR99518.1	0.0
		gi|7271889	86.5±12.5	10.5±3.5	18.0±4.0	Cathepsin L	*F. gigantica*	AAF44675.1	0.0
		gi|21263041	86.5±12.5	10.5±3.5	18.0±4.0	Cathepsin L2	*F. gigantica*	AAM44832.1	0.0
		gi|377823949	86.5±12.5	10.5±3.5	18.0±4.0	Cathepsin L1	*F. gigantica*	AFB77219.1	0.0
		gi|4574304	86.5±12.5	10.5±3.5	15.0±4.0	Cathepsin	*F. gigantica*	AAD23996.1	0.0
		gi|10798511	85.5±13.5	10.5±3.5	14.0±4.0	Cathepsin L1	*F. hepatica*	CAC12806.1	0.0
		gi|211909240	85.5±13.5	10.5±3.5	9.0±4.0	Cathepsin L1D	*F. hepatica*	ACJ12893.1	0.0
		gi|211909242	85.5±13.5	10.5±3.5	9.0±4.0	Cathepsin L1D	*F. hepatica*	ACJ12894.1	0.0
		gi|146147376	70.5±3.5	10.5±3.5	14.0±0.0	Cathepsin	*F. gigantica*	ABQ01982.1	0.0
		gi|41152540	70.5±3.5	10.5±3.5	22.0±0.0	Cathepsin L protein	*F. hepatica*	AAR99519.1	9E-180
		gi|50403821	70.5±3.5	10.5±3.5	16.0±0.0	Cathepsin L1 proteinase	*F. hepatica*	AAT76664.1	0.0
		gi|545734	70.5±3.5	10.5±3.5	18.0±4.0	Cysteine protease	*Fasciola sp.*	AAB30089.1	0.0
		gi|20136379	68.0±15.0	10.5±3.5	26.5±4.5	Cathepsin L, partial	*F. hepatica*	AAM11647.1	0.0
		gi|31558997	67.0±14.0	10.5±3.5	12.0±4.0	Cathepsin L	*F. hepatica*	AAP49831.1	0.0
rFhpCL1	32 (2)	**gi|379991182**	**101.0±29.0**	**14.0±4.0**	**36.5±7.5**	**Cathepsin protein CatL1-MM3p, partial**	***F. hepatica***	**CCA61803.1**	**0.0**
		gi|74765984	101.0±29.0	14.0±4.0	33.5±7.5	Cathepsin L-like proteinase	*F. hepatica*	Q24940.1	0.0
		gi|116488416	97.0±32.0	14.0±4.0	28.5±7.5	Secreted cathepsin L 1	*F. hepatica*	AAB41670.2	0.0
		gi|163310848	97.0±32.0	14.0±4.0	26.0±4.0	Chain A, Crystal Structure Of Procathepsin L1	*F. hepatica*	2O6X_A	0.0
		gi|41152540	95.5±23.5	14.0±4.0	30.0±5.0	Cathepsin L protein	*F. hepatica*	AAR99519.1	9E-180
		gi|50403821	95.5±23.5	14.0±4.0	19.0±0.0	Cathepsin L1 proteinase	*F. hepatica*	AAT76664.1	0.0
		gi|41152538	95.0±23.0	14.0±4.0	28.5±7.5	Cathepsin L protein	*F. hepatica*	AAR99518.1	0.0
		gi|13774082	95.0±23.0	14.0±4.0	30.0±8.0	Cathepsin L-like	*F. hepatica*	AAK38169.1	0.0
		gi|4574304	91.5±26.5	14.0±4.0	21.0±7.0	Cathepsin	*F. gigantica*	AAD23996.1	0.0
		gi|545734	91.0±26.0	14.0±4.0	19.5±3.5	Cysteine protease	*Fasciola sp.*	AAB30089.1	0.0
		gi|7271889	91.0±26.0	14.0±4.0	23.5±7.5	Cathepsin L	*F. gigantica*	AAF44675.1	0.0
		gi|21263041	91.0±26.0	14.0±4.0	23.5±7.5	Cathepsin L2	*F. gigantica*	AAM44832.1	0.0
		gi|146147376	91.0±26.0	14.0±4.0	19.5±3.5	Cathepsin	*F. gigantica*	ABQ01982.1	0.0
		gi|377823949	91.0±26.0	14.0±4.0	23.5±7.5	Cathepsin L1	*F. gigantica*	AFB77219.1	0.0
		gi|108735840	81.0±31.0	14.0±4.0	7.0±0.0	Cathepsin L2	*F. hepatica*	ABG00259.1	7E-167
		gi|211909240	81.0±31.0	14.0±4.0	14.5±7.5	Cathepsin L1D	*F. hepatica*	ACJ12893.1	0.0
		gi|211909242	81.0±31.0	14.0±4.0	14.5±7.5	Cathepsin L1D	*F. hepatica*	ACJ12894.1	0.0

		gi|10798511	80.0±25.0	14.0±4.0	20.5±7.5	Cathepsin L1	*F. hepatica*	CAC12806.1	0.0
		gi|310751866	79.5±25.5	14.0±4.0	27.0±7.0	Cathepsin L-like proteinase	*F. hepatica*	ADP09371.1	0.0
		gi|7271895	77.0±27.0	14.0±4.0	16.5±5.5	Cathepsin L, partial	*F. gigantica*	AAF44678.1	1E-164
		gi|20136379	70.5±6.5	14.0±4.0	32.5±7.5	Cathepsin L, partial	*F. hepatica*	AAM11647.1	0.0
rFhpCL1	28 (3)	**gi|379991182**	**479.0±23.0**	**40.5±0.5**	**62.5±0.5**	**Cathepsin protein CatL1-MM3p, partial**	***F. hepatica***	**CCA61803.1**	**0.0**
		gi|74765984	462.0±10.0	40.5±0.5	58.5±1.5	Cathepsin L-like proteinase	*F. hepatica*	Q24940.1	0.0
		gi|13774082	457.5±7.5	40.5±0.5	52.5±0.5	Cathepsin L-like	*F. hepatica*	AAK38169.1	0.0
		gi|41152538	457.5±7.5	40.5±0.5	53.5±0.5	Cathepsin L protein	*F. hepatica*	AAR99518.1	0.0
		gi|116488416	435.0±9.0	40.5±0.5	43.0±1.0	Secreted cathepsin L 1	*F. hepatica*	AAB41670.2	0.0
		gi|21263041	430.5±6.5	40.5±0.5	40.5±0.5	Cathepsin L2	*F. gigantica*	AAM44832.1	0.0
		gi|7271889	430.5±6.5	40.5±0.5	41.0±1.0	Cathepsin L	*F. gigantica*	AAF44675.1	0.0
		gi|377823949	430.5±6.5	40.5±0.5	40.5±0.5	Cathepsin L1	*F. gigantica*	AFB77219.1	0.0
		gi|4574304	430.5±6.5	40.5±0.5	51.5±1.5	Cathepsin	*F. gigantica*	AAD23996.1	0.0
		gi|163310848	426.0±5.0	40.5±0.5	40.5±3.5	Chain A, Crystal Structure Of Procathepsin L1	*F. hepatica*	2O6X_A	0.0
		gi|41152540	401.5±0.5	40.5±0.5	63.0±1.0	Cathepsin L protein	*F. hepatica*	AAR99519.1	9E-180
		gi|50403821	391.0±2.0	40.5±0.5	25.0±0.0	Cathepsin L1 proteinase	*F. hepatica*	AAT76664.1	0.0
		gi|10798511	378.0±2.0	34.0±16.0	39.5±0.5	Cathepsin L1	*F. hepatica*	CAC12806.1	0.0
		gi|146147376	376.0±0.0	40.5±0.5	27.0±1.0	Cathepsin	*F. gigantica*	ABQ01982.1	0.0
		gi|545734	376.0±0.0	40.5±0.5	33.5±1.5	Cysteine protease	*Fasciola sp.*	AAB30089.1	0.0
		gi|211909240	353.5±3.5	40.5±0.5	33.0±0.0	Cathepsin L1D	*F. hepatica*	ACJ12893.1	0.0
		gi|211909242	353.5±3.5	40.5±0.5	33.0±0.0	Cathepsin L1D	*F. hepatica*	ACJ12894.1	0.0
		gi|7271895	300.5±9.5	40.5±0.5	47.5±0.5	Cathepsin L, partial	*F. gigantica*	AAF44678.1	1E-164
		gi|**310751866**	**245.0±20.0**	**24.0±1.0**	**42.5±0.5**	**Cathepsin L-like proteinase**	***F. hepatica***	**ADP09371.1**	**0.0**
		gi|20136379	241.5±37.5	40.5±0.5	52.5±0.5	Cathepsin L, partial	*F. hepatica*	AAM11647.1	0.0
		gi|108735840	292.5±14.5	40.5±0.5	7.5±0.5	Cathepsin L2	*F. hepatica*	ABG00259.1	7E-167
		gi|31558997	189.5±19.5	40.5±0.5	43.0±1.0	Cathepsin L	*F. hepatica*	AAP49831.1	0.0
		gi|452266	149.0±12.0	40.5±0.5	25.5±4.5	Cathepsin L-like protease	*F. hepatica*	CAA80450.1	8E-124
		gi|19909509	129.5±14.5	29.5±10.5	31.0±1.0	Cathepsin L	*F. gigantica*	BAB86959.1	0.0
		gi|157862759	108.0±12.0	40.5±0.5	46.5±0.5	Cathepsin L, partial	*F. gigantica*	ABV90502.1	0.0
		gi|7271891	100.5±9.5	40.5±0.5	10.0±0.0	Cathepsin L	*F. gigantica*	AAF44676.1	0.0
		gi|107921814	98.5±7.5	40.5±0.5	10.0±0.0	Cathepsin L4	*F. hepatica*	ABF85682.1	2E-172
		gi|167427529	98.5±7.5	40.5±0.5	7.0±0.0	Cathepsin L4, partial	*F. hepatica*	ABZ80401.1	0.0
		gi|195729975	98.5±7.5	40.5±0.5	7.0±0.0	Cathepsin L1	*F. magna*	ACG50798.1	0.0
		gi|38045864	98.5±7.5	40.5±0.5	7.0±0.0	Cathepsin L	*F. gigantica*	AAR08900.1	0.0
rFhpCL1	24 (4)	**gi|379991182**	**148.0±24.0**	**18.5±4.5**	**45.0±1.0**	**Cathepsin protein CatL1-MM3p, partial**	***F. hepatica***	**CCA61803.1**	**0.0**
		gi|163310848	147.5±24.5	18.5±4.5	31.0±1.0	Chain A, Crystal Structure Of Procathepsin L1	*F. hepatica*	2O6X_A	0.0
		gi|74765984	146.5±25.5	18.5±4.5	42.0±1.0	Cathepsin L-like proteinase	*F. hepatica*	Q24940.1	0.0
		gi|116488416	146.5±25.5	18.5±4.5	37.0±1.0	Secreted cathepsin L 1	*F. hepatica*	AAB41670.2	0.0
		gi|7271889	122.5±27.5	18.5±4.5	31.0±0.0	Cathepsin L	*F. gigantica*	AAF44675.1	0.0
		gi|21263041	122.5±27.5	18.5±4.5	31.0±0.0	Cathepsin L2	*F. gigantica*	AAM44832.1	0.0
		gi|377823949	122.5±27.5	18.5±4.5	31.0±0.0	Cathepsin L1	*F. gigantica*	AFB77219.1	0.0
		gi|4574304	122.5±27.5	18.5±4.5	28.0±0.0	Cathepsin	*F. gigantica*	AAD23996.1	0.0
		gi|13774082	122.5±27.5	18.5±4.5	38.0±0.0	Cathepsin L-like	*F. hepatica*	AAK38169.1	0.0
		gi|41152538	122.5±27.5	18.5±4.5	37.0±1.0	Cathepsin L protein	*F. hepatica*	AAR99518.1	0.0
		gi|310751866	119.0±18.0	15.0±1.0	35.5±1.5	Cathepsin L-like proteinase	*F. hepatica*	ADP09371.1	0.0
		gi|50403821	114.5±16.5	18.5±4.5	19.0±0.0	Cathepsin L1 proteinase	*F. hepatica*	AAT76664.1	0.0
		gi|146147376	113.0±18.0	18.5±4.5	25.5±2.5	Cathepsin	*F. gigantica*	ABQ01982.1	0.0
		gi|545734	113.0±18.0	18.5±4.5	24.0±0.0	Cysteine protease	*Fasciola sp.*	AAB30089.1	0.0
		gi|41152540	113.0±18.0	18.5±4.5	35.0±0.0	Cathepsin L protein	*F. hepatica*	AAR99519.1	9E-180
		gi|211909240	105.0±14.0	18.5±4.5	22.0±0.0	Cathepsin L1D	*F. hepatica*	ACJ12893.1	0.0
		gi|211909242	105.0±14.0	18.5±4.5	22.0±0.0	Cathepsin L1D	*F. hepatica*	ACJ12894.1	0.0
		gi|10798511	104.0±15.0	18.5±4.5	28.0±0.0	Cathepsin L1	*F. hepatica*	CAC12806.1	0.0
		gi|108735840	102.5±0.5	18.5±4.5	7.0±0.0	Cathepsin L2	*F. hepatica*	ABG00259.1	7E-167
		gi|20136379	101.0±18.0	18.5±4.5	41.5±1.5	Cathepsin L, partial	*F. hepatica*	AAM11647.1	0.0
		gi|7271895	97.0±6.0	18.5±4.5	22.0±0.0	Cathepsin L, partial	*F. gigantica*	AAF44678.1	1E-164

		gi|31558997	72.0±24.0	18.5±4.5	27.0±1.0	Cathepsin L	*F. hepatica*	AAP49831.1	0.0
rFhpCL1	18 (5)	**gi|379991182**	**270.5±32.5**	**20.5±1.5**	**44.0±0.0**	**Cathepsin protein CatL1-MM3p, partial**	***F. hepatica***	**CCA61803.1**	**0.0**
		gi|74765984	257.0±31.0	20.5±1.5	41.0±0.0	Cathepsin L-like proteinase	*F. hepatica*	Q24940.1	0.0
		gi|13774082	245.5±34.5	20.5±1.5	40.5±0.5	Cathepsin L-like	*F. hepatica*	AAK38169.1	0.0
		gi|41152538	245.5±34.5	20.5±1.5	36.0±0.0	Cathepsin L protein	*F. hepatica*	AAR99518.1	0.0
		gi|20136379	243.5±25.5	20.5±1.5	40.5±0.5	Cathepsin L, partial	*F. hepatica*	AAM11647.1	0.0
		gi|116488416	234.0±30.0	20.5±1.5	36.0±0.0	Secreted cathepsin L 1	*F. hepatica*	AAB41670.2	0.0
		gi|21263041	222.5±33.5	20.5±1.5	34.0±0.0	Cathepsin L2	*F. gigantica*	AAM44832.1	0.0
		gi|7271889	222.5±33.5	20.5±1.5	34.0±0.0	Cathepsin L	*F. gigantica*	AAF44675.1	0.0
		gi|377823949	222.5±33.5	20.5±1.5	34.0±0.0	Cathepsin L1	*F. gigantica*	AFB77219.1	0.0
		gi|4574304	222.5±33.5	20.5±1.5	34.0±0.0	Cathepsin	*F. gigantica*	AAD23996.1	0.0
		gi|163310848	217.0±36.0	20.5±1.5	33.0±0.0	Chain A, Crystal Structure Of Procathepsin L1	*F. hepatica*	2O6X_A	0.0
		gi|41152540	201.0±17.0	20.5±1.5	39.0±0.0	Cathepsin L protein	*F. hepatica*	AAR99519.1	9E-180
		gi|31558997	194.5±27.5	20.5±1.5	26.0±0.0	Cathepsin L	*F. hepatica*	AAP49831.1	0.0
		gi|50403821	184.0±33.0	20.5±1.5	19.0±0.0	Cathepsin L1 proteinase	*F. hepatica*	AAT76664.1	0.0
		gi|146147376	178.0±26.0	20.5±1.5	26.0±0.0	Cathepsin	*F. gigantica*	ABQ01982.1	0.0
		gi|545734	178.0±26.0	20.5±1.5	26.0±0.0	Cysteine protease	*Fasciola sp.*	AAB30089.1	0.0
		gi|310751866	160.0±9.0	18.0±1.0	34.5±0.5	Cathepsin L-like proteinase	*F. hepatica*	ADP09371.1	0.0
		gi|10798511	156.5±6.5	20.5±1.5	28.5±0.5	Cathepsin L1	*F. hepatica*	CAC12806.1	0.0
		gi|452266	154.0±27.0	20.5±1.5	25.5±4.5	Cathepsin L-like protease	*F. hepatica*	CAA80450.1	8E-124
		gi|19909509	149.0±20.0	20.5±1.5	24.0±0.0	Cathepsin L	*F. gigantica*	BAB86959.1	0.0
		gi|211909240	134.5±5.5	20.5±1.5	25.0±0.0	Cathepsin L1D	*F. hepatica*	ACJ12893.1	0.0
		gi|211909242	134.5±5.5	20.5±1.5	25.0±0.0	Cathepsin L1D	*F. hepatica*	ACJ12894.1	0.0
		gi|157862759	105.5±0.5	20.5±1.5	20.0±0.0	Cathepsin L, partial	*F. gigantica*	ABV90502.1	0.0
		*gi|127525	94.0±12.0	3.5±0.5	33.0±0.0	Major outer membrane lipoprotein	*S. marcescens*	P02938.1	4E-46
		gi|7271895	93.0±2.0	20.5±1.5	26.0±0.0	Cathepsin L, partial	*F. gigantica*	AAF44678.1	1E-164
		gi|7271891	89.0±7.0	20.5±1.5	10.0±0.0	Cathepsin L	*F. gigantica*	AAF44676.1	0.0
		gi|107921814	89.0±7.0	20.5±1.5	10.5±0.5	Cathepsin L4	*F. hepatica*	ABF85682.1	2E-172
		gi|167427529	89.0±7.0	20.5±1.5	7.5±0.5	Cathepsin L4, partial	*F. hepatica*	ABZ80401.1	0.0
		gi|195729975	89.0±7.0	20.5±1.5	7.0±0.0	Cathepsin L1	*F. magna*	ACG50798.1	0.0
		gi|38045864	89.0±7.0	20.5±1.5	7.0±0.0	Cathepsin L	*F. gigantica*	AAR08900.1	0.0
		gi|108735840	83.5±5.5	20.5±1.5	7.0±0.0	Cathepsin L2	*F. hepatica*	ABG00259.1	7E-167
rFhpCL1	≤14 (6)	**gi|379991182**	**462.0±16.0**	**40.5±3.5**	**51.0±0.0**	**Cathepsin protein CatL1-MM3p, partial**	***F. hepatica***	**CCA61803.1**	**0.0**
		gi|74765984	436.5±17.5	40.5±3.5	48.0±0.0	Cathepsin L-like proteinase	*F. hepatica*	Q24940.1	0.0
		gi|116488416	432.5±14.5	40.5±3.5	43.0±0.0	Secreted cathepsin L 1	*F. hepatica*	AAB41670.2	0.0
		gi|163310848	427.5±9.5	40.5±3.5	38.0±0.0	Chain A, Crystal Structure Of Procathepsin L1	*F. hepatica*	2O6X_A	0.0
		gi|13774082	422.5±14.5	40.5±3.5	38.5±0.5	Cathepsin L-like	*F. hepatica*	AAK38169.1	0.0
		gi|41152538	422.5±14.5	40.5±3.5	43.0±0.0	Cathepsin L protein	*F. hepatica*	AAR99518.1	0.0
		gi|21263041	418.0±12.0	40.5±3.5	37.0±1.0	Cathepsin L2	*F. gigantica*	AAM44832.1	0.0
		gi|4574304	418.0±12.0	40.5±3.5	37.0±1.0	Cathepsin	*F. gigantica*	AAD23996.1	0.0
		gi|7271889	418.0±12.0	40.5±3.5	37.0±1.0	Cathepsin L	*F. gigantica*	AAF44675.1	0.0
		gi|377823949	418.0±12.0	40.5±3.5	37.0±1.0	Cathepsin L1	*F. gigantica*	AFB77219.1	0.0
		gi|10798511	366.0±27.0	40.5±3.5	29.0±0.0	Cathepsin L1	*F. hepatica*	CAC12806.1	0.0
		gi|41152540	363.0±29.0	40.5±3.5	42.5±1.5	Cathepsin L protein	*F. hepatica*	AAR99519.1	9E-180
		gi|211909240	362.5±24.5	40.5±3.5	28.0±1.0	Cathepsin L1D	*F. hepatica*	ACJ12893.1	0.0
		gi|211909242	362.5±24.5	40.5±3.5	28.0±1.0	Cathepsin L1D	*F. hepatica*	ACJ12894.1	0.0
		gi|146147376	359.5±33.5	40.5±3.5	23.5±0.5	Cathepsin	*F. gigantica*	ABQ01982.1	0.0
		gi|545734	359.5±33.5	40.5±3.5	29.0±1.0	Cysteine protease	*Fasciola sp.*	AAB30089.1	0.0
		gi|50403821	358.5±33.5	40.5±3.5	24.0±1.0	Cathepsin L1 proteinase	*F. hepatica*	AAT76664.1	0.0
		gi|108735840	300.5±22.5	40.5±3.5	7.5±0.5	Cathepsin L2	*F. hepatica*	ABG00259.1	7E-167
		gi|7271895	304.5±18.5	40.5±3.5	30.5±1.5	Cathepsin L, partial	*F. gigantica*	AAF44678.1	1E-164
		gi|20136379	215.5±39.5	40.5±3.5	42.0±1.0	Cathepsin L, partial	*F. hepatica*	AAM11647.1	0.0
		**gi|310751866**	**203.0±35.0**	**27.0±0.0**	**42.0±0.0**	**Cathepsin L-like proteinase**	***F. hepatica***	**ADP09371.1**	**0.0**
		gi|31558997	166.5±38.5	40.5±3.5	32.5±0.5	Cathepsin L	*F. hepatica*	AAP49831.1	0.0

		**gi|19909509**	**123.5±18.5**	**16.0±2.0**	**28.0±0.0**	**Cathepsin L**	***F. gigantica***	**BAB86959.1**	**0.0**
		gi|157862759	109.5±51.5	40.5±3.5	33.0±1.0	Cathepsin L, partial	*F. gigantica*	ABV90502.1	0.0
		gi|452266	106.0±10.0	40.5±3.5	21.0±0.0	Cathepsin L-like protease	*F. hepatica*	CAA80450.1	8E-124
		gi|107921814	105.0±24.0	40.5±3.5	11.0±0.0	Cathepsin L4	*F. hepatica*	ABF85682.1	2E-172
		gi|7271891	105.0±47.0	40.5±3.5	10.0±0.0	Cathepsin L	*F. gigantica*	AAF44676.1	0.0
		gi|167427529	105.0±47.0	40.5±3.5	8.0±0.0	Cathepsin L4, partial	*F. hepatica*	ABZ80401.1	0.0
		gi|195729975	105.0±47.0	40.5±3.5	7.0±0.0	Cathepsin L1	*F. magna*	ACG50798.1	0.0
		gi|38045864	105.0±47.0	40.5±3.5	7.0±0.0	Cathepsin L	*F. gigantica*	AAR08900.1	0.0
